# Targeting the American Market for Medicines, ca. 1950s–1970s: ICI and Rhône-Poulenc Compared

**DOI:** 10.1353/bhm.2014.0075

**Published:** 2014

**Authors:** Viviane Quirke

**Keywords:** anticancer drugs, American medical market, Imperial Chemical Industries, Rhône-Poulenc

## Abstract

The forces that have shaped American medicine include a wide set of interrelated changes, among them the changing research, development, and marketing practices of the pharmaceutical industry. This article compares the research and development (R&D) and marketing strategies of the British group Imperial Chemical Industries (ICI, whose Pharmaceutical Division was spun off and merged with the Swedish company Astra to form AstraZeneca) and its French counterpart Rhône-Poulenc (now part of Sanofi-Aventis) in dealing with the American medical market. It examines how, in the process, the relationship between R&D and marketing was altered, and the firms themselves were transformed. The article also questions the extent to which their approaches to this market, one of the most significant markets for drugs in general, and for anticancer drugs in particular, became standardized in the period of “scientific marketing.”

Modern American medicine is, in the words of a leading scholar in the field, founded upon “a complex nexus of drug and disease, risk and diagnosis, medicine and marketplace.”^1^ However, it was not always so. Over the past fifty years an intimate relationship has developed between the science and business of health care, which has led to a convergence between the priorities of public health authorities and the marketing activities of pharmaceutical firms, and helped to place drugs at the heart of American medical practice in an unprecedented and fundamental way.^2^ This convergence has produced new—largely asymptomatic, flexibly defined—disease categories, based on risk factors such as hypertension, and has contributed to a rise in the incidence of chronic illness. It has created an ever expanding market for drugs, transforming doctor–patient relations and shaping the very experience of patienthood. Because this has been one of the most significant transformations of the past half century, with profound implications not only for American medical practice but for health and medicine more generally, its precise mechanisms and the part played by the different actors deserve to be better understood.

The forces that have fashioned modern American medicine include a wide set of interrelated changes, which range from demography and epidemiology, to political structures and regulation of research and industry, patient activism, and the research, development, and marketing practices of the pharmaceutical industry. The actors involved in these changes therefore count professional associations, government agencies, regulatory authorities, lay advocacy groups, as well as drug companies. In this article I focus on the latter. I explore how two foreign drug companies, the British group Imperial Chemical Industries (ICI, whose Pharmaceutical Division was spun off in 1989, and merged in 1993 with the Swedish company Astra to form AstraZeneca) and its French counterpart Rhône-Poulenc (now part of Sanofi-Aventis), targeted the American market for medicines, helping to construct it through their research and development (R&D) and marketing activities. I also examine how the relationship between these activities was altered, and the firms themselves were transformed in the process.

For this is not only an American story, but one of international circulation of drugs and the knowledge and practices associated with them. This [Other P-655] story shows that the modern pharmaceutical industry is neither monolithic nor static, but—just like modern medicine—flexible and diverse. It also illustrates a striking paradox: that in an age when there were multiple alignments, of public health priorities with those of the pharmaceutical industry,^3^ of pharmaceutical R&D with marketing practices,^4^ and of European with American scientific, technical, and medical knowledge and expertise,^5^ significant differences remained between firms, their drugs, and the diseases they aimed to treat. It is only by studying these differences that one might hope to influence and perhaps modify some of modern medicine’s more worrying trends:^6^ the social and economic problems associated with the growing dependence on drugs to prevent as well as treat disease, and a sense of disappointment with the Therapeutic Revolution,^7^ which has offered treatments more often than it has produced real cures, and has led to a mounting disillusion with modern medicine.^8^[Other P-656]

## Pharmaceutical R&D and the American Medical Market in the Era of “Scientific Marketing”

Between 1990 and 2006 the American market for prescription drugs expanded from $40.3 to $216 billion and continues to grow. Moreover, the United States remain one of the only major world economies to have no explicit price controls, and to allow direct-to-consumer advertising of drugs.^9^ Unsurprisingly therefore, the American medical market has represented an especially attractive target for the European pharmaceutical industry, which, stimulated by increasing opportunities for drug discovery and the mass markets created by national systems of health care in the aftermath of World War II, developed its R&D capabilities and sought to expand its markets abroad.^10^ In the context of alliances and agreements with U.S. multinationals to manufacture antibiotics and other biological drugs, which were often accompanied by the transfer of technical know-how and the diffusion of American management techniques,^11^ European firms tended to align their marketing practices with those of U.S. companies in order to compete on American soil. Because of the growing influence of pharmaceutical marketing on medical practice, this alignment and the rebalancing of firms’ R&D and marketing activities that ensued are worth examining closely and in their wider context, that of a globalized and *globalizing* pharmaceutical industry.

However, while the relationship between clinical research and the marketing activities of drug companies has attracted much attention,^12^ [Other P-657] especially in psychiatry,^13^ and in the United States,^14^ the link between pharmaceutical R&D and marketing has been less well studied, despite the criticisms often aimed at modern drug companies for spending more on marketing in proportion to R&D. Indeed, since the 1980s marketing managers have tended to become involved at an earlier stage of the R&D process, a trend believed by some to be the cause and by others the consequence of the industry’s more recent failure to develop truly innovative medicines, that is, those with a potential to change medical practice in radical and long-lasting ways.^15^

In some countries this trend may have begun long ago, as suggested by a report of the Sainsbury Committee, which was set up by the British government in the 1960s to investigate the relationship between the pharmaceutical industry and the National Health Service, and which found that research and sales promotion were “closely entwined” and had a “profound influence” over each other.^16^ Whatever the variations over time and space, by the late 1980s and early 1990s many industry analysts had begun to argue that a greater integration between R&D and marketing was in fact desirable and perhaps even necessary if pharmaceutical firms were to become innovators and leaders within the medical marketplace.[Other P-658]

The changing process and context of drug discovery had already started to offer companies a number of opportunities for greater integration. One of these was “rational drug design” (i.e., drug discovery based on fundamental knowledge about particular disease targets), which enabled the intervention of marketing managers early on in the innovation process.^17^ Another was the need to “master the art of dealing with regulatory agencies.”^18^ Following the thalidomide tragedy, these agencies became major gatekeepers to the market for medicines, not only nationally, but also globally, as steps to harmonize new drug approval procedures were taken among developed countries. New, more stringent drug safety legislation not only posed a challenge to companies’ marketing departments, but also, by increasing development times for novel drugs (and therefore also the ratio of development to research costs), altered the balance between the R and D in R&D departments, once again leading to an earlier involvement of marketing staff in the innovation process.^19^ Companies that adopted a targeted approach to drug development and/ or nurtured their relationship with the regulatory authorities were therefore more likely to innovate and become market leaders.^20^

Such companies included ICI. However, while ICI had turned to the American market for medicines as an inspiration for its research strategy as early as the 1950s, adopting a many-pronged approach to R&D in all the therapeutic areas in which it ventured, including cancer, its French counterpart, Rhône-Poulenc, did not appear to consider the American market for its anticancer drugs seriously until the 1970s. Although its cancer chemotherapy program was well developed by then, and the French firm had successfully launched its psychiatric drug chlorpromazine onto the American market in the 1950s, its cancer research program had been aimed first at the domestic, then at the wider European market. When the American market at last appeared to be considered, the way in which it was targeted mirrored the approach adopted at home, privileging collaborative relations with clinical researchers in order to influence medical opinion and—eventually—smooth the path to obtaining market authorization from the FDA. In this article I therefore question the extent [Other P-659] to which approaches to the American medical market were truly standardized in the era of “scientific marketing” (defined as well-organized marketing programs, based on standardized marketing practices, using a quantitative approach to marketing problems, and requiring a specialized sales force). Scientific marketing had gathered momentum in the United States in between the wars, spreading after World War II to Europe, often via American firms of management consultants.^21^ However, a comparison between ICI and Rhône-Poulenc shows the limits to this process of Americanization, as well as the importance of national traditions, which have persisted within the global pharmaceutical industry.

This comparison also demonstrates that different therapeutic areas have specific characteristics that need to be taken into account in studies of pharmaceutical R&D and marketing. Because of its biological complexity, cancer involves a greater technical risk for firms seeking to generate major new treatments than, say, heart disease.^22^ This may explain why governments have tended to be more closely involved in R&D relating to cancer, instigating applied research programs into cancer therapies in state-funded laboratories, and initiating nationwide campaigns for cancer prevention and control.^23^ Hospital doctors have also tended to be more proactive, helping to establish large-scale, cooperative trials as a key component of modern biomedical practice,^24^ and playing a major role in improving the modalities and usage of cancer chemotherapy.^25^ However, it was not always so. Until the early twentieth century, few were the physicians who showed interest in the study of cancer, and rare were the medical institutions that took on patients deemed “incurable.” Even after attitudes had begun to change as a result of the creation of scientific societies and medical institutes dedicated to the study of malignant disease and the care [Other P-660] of its sufferers, the prognosis for many cancers remained so dire that the size of the potential market and degree of unmet therapeutic need would have been particularly difficult to assess, this at a time when deaths from cancer were felt to be rising to epidemic levels.^26^ Nevertheless by the late 1980s the potential market for anticancer drugs was described as medium, situated in terms of market size between type 1 diabetes at the lower end and obesity and depression at the higher end, and in terms of therapeutic need between glaucoma at the lower end and Alzheimer’s and AIDS at the higher end.^27^ Today the chemical and biological agents used to treat cancer amount to a multibillion-dollar industry.^28^ Consequently, more perhaps than many other types of drugs, the first successful anticancer drugs could be said to have “created their own market worth.”^29^ I therefore begin with a brief overview of the early history of cancer chemotherapy, focusing on the American context, which played an important part in the development of this particular form of cancer treatment.

## Constructing the American Market for Anticancer Drugs

How is a market defined, how is it targeted, and how, in turn, is it constructed? Nowhere are these questions more relevant perhaps than in connection with the American market for anticancer drugs. Indeed, this market could be said not to have existed until the first anticancer drugs were launched by American pharmaceutical firms in the United States, where organized science, mobilized on a large scale in order to wage “war on cancer,” provided a fruitful context for their development and use.^30^ In the early period in the history of cancer chemotherapy covered by this article (ca. 1950s–1970s), much of R&D—both public and [Other P-661] private—focused on leukemia, which did not respond well to surgery or radiotherapy, and therefore provided opportunities as well as incentives for novel forms of treatment. The fact that leukemia, more specifically childhood leukemia, became the first malignant disease to be “cured” with drugs helped to establish chemotherapy as a viable and valuable approach in the treatment of cancer.

This early period in the history of cancer chemotherapy occurred in three broadly defined phases. Although they overlapped with one another, each phase represented a change in the style and scale of research: from largely local experimentation (in the 1940s–1950s) to national and then international mass-screening programs (1950s–1960s), and finally global multicenter trials (1960s–1970s). Spurred on by the creation in 1955 of the U.S. Cancer Chemotherapy National Service Center (CCNSC), which invited foreign firms to join its mass-screening program, European companies started targeting the American market for anticancer drugs mainly in the second phase, that is, the 1950s and 1960s. However, the hurdles they needed to overcome were considerable. Not only were firms required to compete with their American counterparts on their home ground, they also had to adapt themselves to this particular market in order to satisfy the preferences of American clinicians as well as the requirements of the FDA.

## Phase 1: Discovering and Experimenting with Anticancer Drugs (1940s–1950s)

This first phase in the development of cancer chemotherapy was largely experimental, characterized by a trial-and-error approach and with benefits to patients seemingly haphazard and uncertain. In this first phase four main groups of anticancer drugs were discovered, which came to be distinguished from one another by their mechanism of action: (1) alkylating agents (at this early stage mainly nitrogen mustards), (2) natural and synthetic hormones, (3) antimetabolites, and (4) cytotoxic antibiotics. From these different groups would emerge a coherent, and eventually fruitful, approach to cancer treatment: cancer chemotherapy. But its history is far from linear or predictable.

Indeed, while the use of surgery to treat cancer goes back a long way, the history of radiotherapy began only in the 1910s and 1920s, and that of cancer chemotherapy is more recent still. It resulted from the convergence between fundamental discoveries and empirical research, often in areas unrelated to cancer, and was facilitated by the development of laboratory techniques as well as animal models for testing anticancer [Other P-662] drugs.^31^ Although at the end of the eighteenth century the English doctor Thomas Fowler (1736–1801) had prepared and sold a hydroalcoholic solution of potassium arsenite, which was used to treat a variety of conditions, including leukemia,^32^ the origins of cancer chemotherapy are usually traced to World War II, with the discovery made at Yale University by Louis Goodman and Alfred Gilman in 1942 that nitrogen mustards, which were being studied because of fears of renewed poison gas attacks, had the ability to shrink tumors of white blood cells in mice. The compound code-named HN3 (2,2,“2”-trichloroethylamine) was then tried in a patient suffering from a lymphatic tumor in the chest and face that had become resistant to radiotherapy.^33^ The tumor shrank and for a time the patient’s condition improved. Although the tumor stopped responding to treatment and started growing again, leading to the patient’s death, the initial results had been dramatic enough to warrant further studies. Because of the agreement that existed between the American and British governments to exchange scientific and technical information, research on nitrogen mustards and related compounds spread quickly to other academic and industrial research centers, not only in the United States, but also the United Kingdom, including the Chester Beatty Institute in London and the Medicinal Section of ICI’s Dyestuffs Division in Blackley, near Manchester.^34^ Later, knowledge about the mechanism of action of nitrogen mustards, that is, their alkylating action,^35^ guided the search for other compounds with similar anticancer properties. One of them was tretamine, discovered almost simultaneously in 1950 by ICI and by American Cyanamid’s Lederle Division, which collaborated with Joseph Burchenal and Chester Stock at the Sloan Kettering Institute for Cancer Research in New York. Tretamine was soon followed by thiotepa, made and marketed by American Cyanamid for use in ovarian and bladder tumors,^36^ while several alkylating drugs developed by researchers at the Chester [Other P-663] Beatty Institute in the 1950s (busulphan, chorambucil, and melphalan) were marketed by the British firm Burroughs Wellcome.

Meanwhile, sex hormones and synthetic analogues had been tried in various cancers of the sex organs, in particular of the breast and of the prostate. The link between hormones and cancer had been known at least since 1916,^37^ but their usage in the treatment of cancer depended on their isolation, purification, and chemical determination, which were not achieved until the 1930s in the case of sex hormones. One such hormone was the follicular hormone, which was prepared by the Roussel Laboratories, a French company specializing in biologicals, and supplied to Antoine Lacassagne at the Institut du Radium in Paris. Using this hormone, Lacassagne was able to show a direct link between estrogens and the appearance of breast cancer in mice.^38^ But natural estrogens were difficult to obtain in the vast quantities needed for large-scale experiments. This changed when E. C. (later Sir Charles) Dodds and his collaborators at the Middlesex Hospital in London discovered that the synthetic compound stilboestrol had estrogenic properties.^39^ Other synthetic substances were soon found to have similar estrogenic activity, such as triphenylethylene. These substances, which could not only be mass produced, but also be modified to obtain analogues with antiestrogenic action, became compounds of choice for studies in Britain and elsewhere. In 1939 Charles Huggins of the University of Chicago successfully treated cases of prostate cancer with stilboestrol (known as diethylstilbestrol in the United States),^40^ and by 1950 a cooperative study had shown that the synthetic estrogen was effective in delaying the progress of this type of malignant [Other P-664] disease.^41^ However, breast cancer proved more complex to treat, as it could be either inhibited or stimulated by administration of estrogen. Nevertheless, as early as 1944, Alexander Haddow of the Chester Beatty Institute and his collaborators demonstrated that stilboestrol and some of its analogues could be beneficial in the treatment of breast cancer, thus paving the way for what would become the adjuvant chemotherapy of breast cancer.^42^ Other hormones and their synthetic analogues would later also be used to treat cancer in the organs that they control, such as the adrenal cortical hormones ACTH and cortisone to treat cancer of the lymphocytes (e.g., leukemias).^43^

A third approach that also originated in World War II, and would not only produce useful anticancer drugs, but also—perhaps more significantly—modify people’s perception of cancer as an incurable disease, was the development of antimetabolites. This approach resulted from the understanding of the mechanism of action of the antibacterial drug sulphanilamide. In 1940, D. D. Woods, who had joined Paul Fildes’s group at the Middlesex Hospital, showed that sulphanilamide acted by a process called “competitive antagonism.” Indeed, the chemical structure of sulphanilamide resembled that of *p*-aminobenzoic acid (PABA), a substance necessary for the growth of certain species of bacteria. When these bacteria took up sulphanilamide instead of PABA, they failed to grow and died.^44^ Although the principle of competitive antagonism had been known for quite a while, its use to explain the antibacterial properties of sulphanilamide suggested the possibility of selectively poisoning bacteria without harming their host, and inspired the search for drugs with a similar action, against not only microorganisms, but also other cells, including cancer cells.^45^ Indeed, soon after Woods and Fildes’s discovery, it was shown that PABA is a component of folic acid, which is a vitamin present in green leafy vegetables such as spinach, and is useful in the treatment of certain types of anemia. The idea of selectively poisoning white blood cells, which proliferate abnormally in leukemia, by administering [Other P-665] “antimetabolites” (i.e., antagonists of substances such as folic acid) was therefore pursued by a number of research laboratories.

One of these was Richard Lewisohn’s laboratory at the Mount Sinai Hospital in New York, where he had devised a method for preserving blood for transfusion in 1915. Having turned his attention to the problem of cancer, in 1944 Lewisohn showed that the milk fermenting organism, *Lactobacillus casei*, which contained a form of folic acid, was capable of reducing mammary tumors in mice. American Cyanamid had processed blood and derivatives during the war, and afterward diversified into vitamins and related products.^46^ Spurred on by Lewisohn’s work, in 1946 one of its teams of researchers, led by the Indian biochemist Yellapragada SubbaRow, succeeded in working out the structure and synthesis of folic acid.^47^ Analogues of the substance were made and sent not only to Lewisohn, but also to SubbaRow’s former colleague at Harvard, the pediatric pathologist Sidney Farber, who administered them to children suffering from leukemia in the Children’s Medical Center in Boston. However, in a number of patients, the folic acid analogues had the opposite effect to that intended, that is, they accelerated the progress of the disease. With Farber’s clinical findings guiding their chemical work, SubbaRow’s team therefore synthesized a further series of compounds, which included aminopterin, followed by methotrexate in 1947. The publicity that surrounded Farber’s early successes with these drugs was such that it inspired similar attempts elsewhere, including Paris, where the French clinician Jean Bernard used antifolates supplied by Lederle in his pediatric ward at the Hérold Hospital.^48^ The therapeutic regimen that was developed in association with antifolates, and owed much to one of Farber’s former associates, Donald Finkel, later medical director at St. Jude’s Children Hospital in Memphis, would eventually produce cases of remission and even cure from this hitherto largely fatal disease.^49^

Meanwhile, a different though related path to the development of antimetabolites was being pursued by George Hitchings, working at Tuckahoe, New York, in the American subsidiary of the British drug company Burroughs Wellcome. Hitchings’s previous studies at Harvard had involved the quantitative estimation of purines, one of the nucleic [Other P-666] acid bases, as well as the purification of the antianemia principle in liver. His further studies on the topic, carried out at Western Reserve, had led to the identification of folic acid. When he joined Burroughs Wellcome in 1942, like other researchers in the field, Hitchings reasoned that the search for antimetabolites might lead to compounds with selective toxicity. However, unlike others, Hitchings chose to concentrate on nucleic acids. Although it was generally understood that bacteria and other microorganisms rely on the rapid biosynthesis of nucleic acids for their survival, this was an area of research that then, before the structure of DNA and its precise role in cell division were known, was the sort of “quiet backwash where a small group might work relatively undisturbed by the pressures of intensive competition.”^50^ A possible approach consisted in synthesizing both purine and pyrimidine analogues, and testing their inhibitory activity and the reversibility of this action in a suitable experimental system. Once again, *Lactobacillus casei* (which grows on the pyrimidine and purine bases of nucleic acids) provided such a system. A consequence of this—at once empirical and fundamental—approach to drug development was that disease targets were not defined in advance. Rather, as Hitchings put it, they “were bound to follow wherever [their] thoughts and anti-metabolites led [them].”^51^ Nevertheless, once the first successes in the treatment of leukemia had been achieved with antifolates, the study of cancer became a major focus for the research team, now joined by Gertrude Elion, with whom Hitchings would later share the Nobel Prize for Physiology or Medicine. In 1948 Elion prepared 2:6-diaminopurine, which produced spectacular remissions in a number of leukemia sufferers.^52^ This had the effect of strengthening the link that already existed between Hitchings and Burchenal, the head clinician at Sloan Kettering, which thereafter funded their collaborative research program, leading to further synthetic work with purines. In 1951 Hitching’s group synthesized 6-mercaptopurine (6MP), which proved to be effective in sarcomas as well as several types of leukemia in mice, and which produced complete remissions in about one-third of children whose leukemias had become resistant to methotrexate. Soon afterward 6MP was approved for use in leukemia by the FDA, in 1953.^53^[Other P-667]

Unsurprisingly, antibiotics, which constituted one of the principal pillars of the second (pharmacological) Therapeutic Revolution that had begun in the 1930s with the invention of the sulphonamides, were also tried in cancer.^54^ In the search for new antibiotic substances, vast numbers of molds and microorganisms were collected and screened for a wide range of activity, and discoveries often occurred simultaneously in different laboratories.^55^ In 1949, a researcher at Göttingen University in Germany read a report that the antibiotic Actinomycin A, which had been isolated by Selman Waksman at Rutgers University, was active in one of the Sloan Kettering Institute’s tumor screens. The German researcher therefore sent a related strain, Actinomycin C, to the Bayer Institute for Experimental Pathology in Elberfeld. There it was also found to inhibit tumor growth in mice, hence it was tried in patients with lymphatic tumors.^56^ The news got back to Waksman, and he sent some Actinomycin C to Farber, who gave it to a boy suffering from Wilm’s tumor in the kidney, with metastases in the lungs. Although the boy died three weeks later, the postmortem revealed that his metastases had disappeared. The results were encouraging enough to justify the search for related antibiotics, and in 1953 Waksman isolated Actinomycin D, which was shown not only to cure Wilm’s tumor, but also to be effective in several other types of cancer.^57^ A decade later two other antibiotics with similar cytotoxic activity were developed independently but simultaneously by laboratories in France, Italy, and Japan: daunomycin and bleomycin.^58^

## Phase 2: The Mass Screening and Testing of Anticancer Drugs (1950s–1960s)

The mass screening for antibiotics fitted well with the approach that was becoming predominant in the search for anticancer agents, namely the mass screening of natural as well as chemical substances for antitumor activity. In 1935, Murray Shear had created one of the first drug screening programs under the auspices of the Office of Cancer Investigations at the U.S. Public Health Service, which two years later merged with the Laboratory of Pharmacology of the National Institutes of Health to form the [Other P-668] National Cancer Institute (NCI).^59^ In 1947, the Sloan Kettering Institute developed its own independent large-scale screening program, inviting submissions of materials for screening from the top American chemical and pharmaceutical firms: not only American Cyanamid and Lederle, which we have already encountered, but also DuPont, Eastman Kodak, Dow Chemical, and Union Carbide on the chemical side, and Parke-Davis, Merck, Sharp & Dohme, Lilly, Abbott Laboratories, Pfizer, Upjohn, and Searle on the pharmaceutical side. Sloan Kettering also sent invitations for submissions to universities, hospitals, and other public institutions in the United States and beyond, in England, Ireland, France, Germany, Switzerland, India, and Australia.^60^ Perhaps because of what was seen as duplication of effort between the NCI and Sloan Kettering, but also because of the public’s opposition to drug testing in cancer and the fact that so far only two drugs had been taken to clinical trial before being abandoned as a result of excessive toxicity, the NCI’s screening program was dissolved in 1953.^61^ However, in the same year, under the influence of a powerful lobby composed of Cornelius Rhoads, director of the Sloan Kettering Institute, Mary Lasker of the American Cancer Society, and Sidney Farber of the Children’s Cancer Research Foundation, Congress directed the NCI to begin a new research program focusing on the chemotherapy of acute leukemia. Within two years, the publicity surrounding Farber’s first achievements with methotrexate encouraged Congress to push for an even stronger screening program within NCI, embodied in the CCNSC formed in 1955.

The CCNSC continued Sloan Kettering’s practice of screening compounds offered by public bodies and private companies under “commercial discreet agreements,” but the scale of the screening facilities provided by the CCNSC was soon ten times greater than that of Sloan Kettering.^62^ In addition, the CCNSC provided access to supplies of laboratory mice and human and animal tumors for drug testing, as well as clinical testing facilities.^63^ When it began in 1955, six anticancer drugs had been approved for clinical use in the United States, and they were either antimetabolites or alkylating agents.^64^ At first the CCNSC therefore focused on synthetic compounds, although some antibiotics were also included in the screens.[Other P-669] Three years later, in 1958, plants started being included in the CCNSC’s screens, which would eventually lead to the discovery of the antitumor properties of the Vinca alkaloids and the bark of the Pacific yew.^65^ By the 1980s, as the number of anticancer drugs emanating from the synthetic chemical industry declined, those of natural origin (fermentation products, and animal or plant materials) grew in importance. A large proportion of them came from Europe, where they were developed under the aegis of the European Organization for Research and Treatment of Cancer, created in 1957 in response to the Common Market as well as the CCNSC.^66^

## Phase 3: Targeted Drug Development and Cooperative Multicenter Trials: The “Final Push” (1960s–1970s)

The CCNSC’s intensive targeted drug development program was mirrored in large-scale clinical trials that enabled the systematic evaluation of novel drugs, and galvanized drug companies into setting up formal cancer research programs, not only in the United States, but also abroad.

However, the value of chemotherapy in relation to cancer was still hotly debated and remained to be proven. It was not until the new drugs and clinical trial methodology were combined in a third, “final push” that chemotherapy would become established, eventually overtaking other forms of therapy for leukemia in particular, and malignant disease more generally.^67^ Although its early development had owed much to institutions like Sloan Kettering, during this third phase in its history, which started around 1962, clinicians came to the fore, such as Frei and Freireich, who helped to establish combination therapy in what was retrospectively acknowledged as a “landmark trial” at the National Institutes of Health’s Clinical Center in Bethesda, Maryland.^68^ In this third phase industry also played a major role in collaboration with the clinic, and its drugs and associated therapeutic regimens would help to shape the market for anticancer drugs. It is this third phase, and the contribution made by ICI and Rhône-Poulenc, on which the rest of this article focuses.[Other P-670]

As Weatherall has argued, advances in treating cancer have tended to come not from research directed specifically against cancer, but from discoveries made in quite different fields that have converged toward cancer.^69^ Industry, where disciplinary boundaries can perhaps more easily be broken down and discoveries in one therapeutic area can more readily be applied to another, therefore became a fertile site for cancer research. However, the convergence described by Weatherall also meant a proliferation of potential anticancer drugs, in a market where the top American chemical and pharmaceutical firms had already begun jostling for position. As we shall see with the examples of ICI and Rhône-Poulenc, although both firms are comparable in that they were chemical groups and had similar origins, their different historical trajectories led them to approach the American medical market in different ways. Hence I begin with brief descriptions of their histories, before turning to their cancer research programs and marketing strategies.

## ICI, the Beta-Blockers, and the American Market for Drugs

Created in 1926 and inspired by the German chemical giant IG Farben,^70^ almost from the very start ICI’s global (at first mainly “imperial”) outlook included an American perspective. Indeed, under a Patents and Process Agreement signed in 1929, and which lasted until 1949, ICI developed a close but at times strained relationship with the American group DuPont.^71^ This agreement, which had the purpose of dividing the world markets between the two groups and making it easier for them to compete with IG Farben, led to extensive sharing of scientific and technical information. However, in the pharmaceutical field, which ICI entered earlier than its American partners with the creation of a Medicinal Section within its Dyestuffs Division in 1936, the agreement restricted ICI’s freedom of action to sign agreements with other firms, for instance Squibb, which developed deep-fermentation methods to manufacture penicillin in World War II.^72^

During the conflict, ICI became the British government’s largest industrial agent, playing a crucial role in Anglo-American projects to develop not only the atom bomb and radar, but also synthetic antimalarials, penicillin, and anticancer agents. By 1944, this had led to the creation of a [Other P-671] separate Pharmaceutical Division. In 1953, plans were made for a new pharmaceutical research center near Manchester, Alderley Park, which was envisaged as a “centre for speculative chemotherapeutic research.”^73^ Although no clear instructions were given on the amount that should be spent, research expenditure at ICI’s Pharmaceutical Division had so far represented about 10 percent of its turnover, a figure similar to that “applied to IG Farben (prewar) and USA pharmaceutical firms [postwar].”^74^ American firms provided a model for ICI in other ways too. The creation in 1949 of ICI’s central research laboratory near Welwyn was inspired by DuPont’s Experimental Station at Wilmington, where scientists were given the freedom to work on fundamental research projects.^75^ Wilmington also provided a model for Alderley Park, where, from the very beginning, cardiovascular function was a major focus of research, as part of a wider program to study chronic diseases. This program was based on quantitative evidence drawn from epidemiological data compiled by ICI’s medical department, as well as on surveys of the activities of American firms and research centers, which suggested to ICI promising new fields for pharmaceutical R&D (both from the point of view of feasibility and profitability).^76^ Although there was uncertainty as to the outcome of this research program, which remained speculative at this stage, in other areas, such as anesthetics, ICI already had an innovative and marketable product in Fluothane, and with it began targeting the American market as early as 1956.^77^

Beta-blockers, which were discovered in the early 1960s, were the main outcome of ICI’s cardiovascular research program. They had been developed using a guided or targeted approach (i.e., identifying therapeutic needs and choosing suitable molecular targets to respond to these needs from the very start of the research project, rather than simply screening compounds in search of active compounds) sometimes referred to as [Other P-672] “rational drug design.”^78^ The beta-blockers also led ICI to combine its R&D and marketing activities in new ways. Last but not least, they represented one of the firm’s earliest attempts to develop a close working relationship with the FDA, so I will say a little more about them here, before moving on to ICI’s cancer research program.

When James Black, a physiologist with medical training, arrived at Alderley Park in 1958 to work on coronary artery disease, he brought with him something new, which was to provide ICI with an edge over their competitors in Britain and abroad: Raymond Ahlquist’s (of the University of Georgia at Atlanta) theory of alpha- and beta-receptors, which suggested to Black that it might be possible to treat angina-pectoris by blocking the beta-receptors responsible for increased heart rate.^79^ Within a few months of his arrival, he had read in the most recent issues of the *Journal of Pharmacology and Experimental Therapeutics* about the American firm Eli Lilly’s new compound, dichloroisoproterenol (DCI), which had the kind of properties he was looking for. Eli Lilly, who had developed DCI as a bronchodilator, but found it to have unwanted cardiac side effects, had not seen the compound’s potential in cardiovascular medicine. Under Black’s direction, ICI did, and promptly developed a derivative of DCI, pronethalol, as the first clinically useful beta-blocker. The drug underwent small-scale trials in 1961, and was launched in 1963 under the name Alderlin (after Alderley Park).

**Figure 1:**
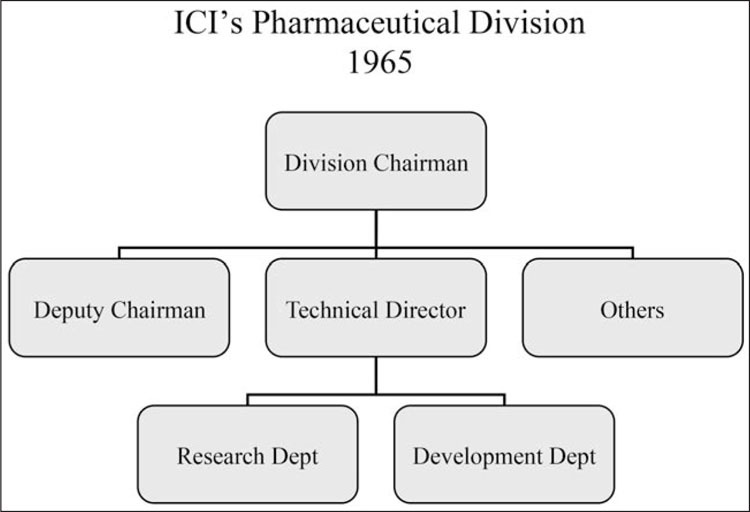
Organigram of ICI’s Pharmaceutical Division in 1965. Source: AZ DO 770, Director’s Secretary’s Dept., May 1965.

In 1962, Black’s team was joined by R. G. Shanks, a physiologist who had spent a year with Ahlquist at the University of Georgia. Synthesis of analogues continued, in order to provide Alderlin with wide patent coverage and protect its position on the market against possible competitors. In the process, a compound with greater activity than Alderlin was discovered. It was propranolol, which was to replace Alderlin after it had been found to cause tumors in mice. The development of propranolol (launched as Inderal in 1965) prompted two organizational innovations at ICI: (1) the Submission to the Committee of the Safety of Drugs (the CSD, established in 1963) and (2) the Development Programme, which coincided with the hitherto separate Research and Development Departments coming together under the responsibility of a single director, the technical director (see Figure 1).^80^[Other P-673]

The authors of the Inderal Development Programme therefore included not only Robin Shanks of the Research Department, but also members of the Development Department, the Homes Sales Control Department, the Medical Services Department, and the Overseas Convenor. As well as producing market evaluations and sales estimates, one of its main purposes was to plan clinical trials of propranolol in “all major markets.”^81^ The largest of these was the United States, where ten trials were being organized in six different medical conditions. Publication plans were included in the program, with articles to be sent to scientific and medical journals, and papers on the pharmacology of Inderal to be presented at the meeting of the American Society for Pharmacology and Experimental Therapeutics in Lawrence, Kansas, in August 1964. Further laboratory and toxicity studies were also planned, to meet the FDA’s requirements (including a special investigation of the teratogenic potential of Inderal) as well as make a submission to the CSD. Although submissions to the CSD were not yet compulsory for market launch in the United Kingdom, ICI’s Medical Department nevertheless decided to prepare such a submission as a useful “exercise,” which would later help them in preparing FDA submissions, including in the cancer field.[Other P-674]

ICI’s strategy bore fruit: in 1969, the company obtained clearance from the Committee for the Safety of Medicines (the CSM, successor to the CSD) to market Inderal in hypertension, followed in 1973 by the FDA’s approval of the drug as an antihypertensive agent. These events helped to establish the use of beta-blockers in the treatment of hypertension, while helping to transform hypertension into a chronic disease amenable to long-term drug treatment.^82^ They paved the way for atenolol (Tenormin), ICI’s best-selling hearty drug, which within ten years of launch in 1976 generated sales worldwide of about £500 million, nearly 40 percent of which were made in the United States.^83^

With the beta-blockers, ICI had evolved a targeted approach to drug discovery as well as a many-pronged approach to R&D and marketing, in which both functions were closely entwined. Perhaps because of ICI’s late entry into pharmaceuticals, it had to rely heavily on its medical department, which played a pivotal role at the junction between the two functions, not only by identifying therapeutic needs early on in the R&D process, but also interacting with the regulatory authorities, such as the CSD/CSM, and most important, as we will see in the case of cancer, with the FDA.

## ICI and Cancer Research

In contrast to its cardiovascular research program, ICI’s cancer program was hesitant and patchy, as both the feasibility and profitability of research into cancer were most uncertain, and it would be a long time before industry could command the high prices for its cancer drugs that we know today.^84^ However, the company’s interest in cancer predated its interest in heart disease. When triphenylethylene was found to act as a synthetic estrogen, Arthur Walpole, a biologist who had joined ICI’s Medicinal Section in 1938, began some exploratory work using the substance in the treatment of breast cancer in collaboration with Edith Paterson at the Christie Hospital in Manchester and Alexander Haddow at the Chester [Other P-675] Beatty Institute in London.^85^ As we saw earlier, the compounds (known as “nitrogen mustards”) had been shown to inhibit the growth of blood and lymph tumors. Walpole therefore began to investigate them in other cancers, once again in collaboration with the nearby Christie Hospital. Meanwhile, a parallel study relating to cancer had developed within ICI, involving antimetabolites. During the war, the search for sulphonamides and synthetic antimalarials for use on the battlegrounds of Europe and the Far East had led to the study of a variety of compounds. Following the discovery that ICI’s novel antimalarial drug Paludrine was converted in the body to cycloguanine, an active metabolite that interferes with purine biosynthesis,^86^ and spurred by the announcement that Burroughs Wellcome’s drug 6MP was effective against leukemia, the search for antimetabolites began at ICI under the leadership of Frank Rose, who had run its antimalarial program during the war. Rose became research manager of the Chemistry Department in 1954, while remaining involved in bench work. As well as the search for alkylating agents, synthetic estrogens, and antimetabolites, Rose also encouraged investigations into carcinogenesis (which his biographers observed was a rare interest for people working on cancer chemotherapy at that time).^87^

**Figure 2:**
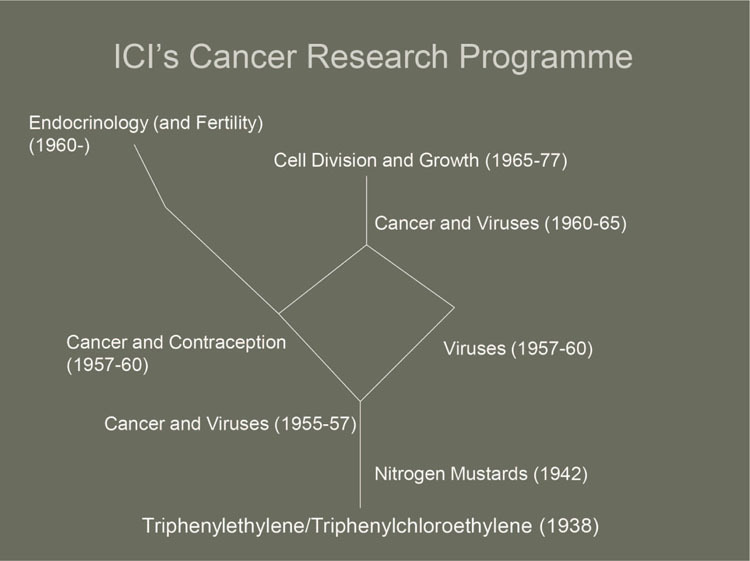
ICI’s cancer research program.

At first, ICI’s approach to cancer was therefore largely empirical, involving the synthesis of analogues of compounds that had known antitumor properties, without a formal cancer research program. However, once plans had been made to build a pharmaceutical research center at Alderley Park, cancer became a team project at ICI in 1955, that is, the year of the creation of the CCNSC. The project was titled Cancer and Viruses: Antibacterials,^88^ and its team leader was biologist E. Weston Hurst. Alderley Park opened in 1957, and between 1957 and 1960 Cancer and Viruses separated into two different projects (see Figure 2).

During that time Cancer was merged with a new project to find an oral contraceptive, led by Arthur Walpole. Then, in 1960, the discovery [Other P-676] of the natural antiviral substance interferon, and ICI’s involvement in its study in collaboration with the Medical Research Council,^89^ led to Viruses and Cancer coming together again. Oral Contraception therefore split away from Cancer, with Walpole working in parallel on both projects. His involvement in the Oral Contraception project (which in 1963 was renamed Endocrinology, and later Fertility) would ensure that breast cancer remained an important research focus for his team.

It was within this Oral Contraception project that tamoxifen (Nolvadex), a triphenylethylene analogue, was synthesized and developed, originally as a contraceptive pill.^90^ However, on the tamoxifen patent (1963),^91^ Walpole also included “control of hormone-dependent tumours” as a potential area of application. As we saw earlier, there was a long tradition of synthetic estrogens being used in the treatment of hormone-dependent cancers. But unlike many other similar compounds, tamoxifen had unique [Other P-677] characteristics—in particular its lack of androgenic properties—which encouraged its adoption by the medical profession,^92^ and despite its initially poor market prospects helped to transform it from a quasi-orphan to a blockbuster drug in a relatively short period.

**Table 1:** U.K. Major Branded Products

	Annual sales (NHS level) (£)	Cost of 1 week’s treatment (£)
Provera 100 (progestogen, Upjohn)	50,000	3.15
Masteril (anabolic/androgen, Syntex)	45,000	1.35
Deca-durabolin (anabolic/androgen, Organon)	35,000	0.75
Durabolin (anabolic/androgen, Organon)	28,000	0.50
SH420 (progestogen, Schering)	27,000	0.70
Source: AZ PH 19597 B, Nolvadex Development Programme, June 1971.		

A first stage in this transformation involved gaining approval for clinical trials in cancer, which Walpole obtained from the CSM in 1969. Then, in 1971, the Nolvadex Development Programme was drawn up,^93^ which would play an important part in the drug’s trajectory from bench to bedside.^94^ As well as trials in contraception, it included plans for cancer trials, which had begun on a small scale at the nearby Christie hospital,^95^ before being extended to large-scale, multicenter trials. In addition to making plans for clinical trials, the Nolvadex Development Programme assessed the competitive situation. It showed that a number of chemotherapeutic treatments for hormone-dependent breast cancers were already in existence, each commanding almost equal shares of the market (Table 1).

The Development Programme also assessed the “position in North America,” and expressed concerns for tamoxifen’s fate on the other side of the Atlantic. These concerns were based on Ayerst’s earlier rejection [Other P-678] of ICI’s offer of Nolvadex for the U.S. market, as well as suspicions that the FDA would take an unfavorable view of the use of tamoxifen in breast cancer.^96^ Such concerns and suspicions were aggravated by the recent finding that, contrary to what might be expected from the laboratory studies in rats, tamoxifen did not work as a contraceptive pill in women; quite the contrary, it helped to stimulate ovulation in women who had trouble conceiving. The market for a fertility drug was small, as seemed the market for an anticancer drug, partly due to the poor prognosis associated with the disease. Despite growing clinical evidence of the usefulness of tamoxifen in breast cancer, the Marketing Department produced very low sales estimates of two kilograms for initial sales stock. This prompted the following comment from Dora Richardson, the ICI chemist who had synthesized tamoxifen, and was the author of an unpublished history of the drug: “from the figures we only envisaged treating dead people!”^97^ Consequently ICI’s Main Board decided to close down the Program, but tamoxifen’s champion, Walpole, threatened to resign, and as a result it was saved.

In 1970 anticipated sales had been only £100,000 annually. By 1980, tamoxifen was making £30 million for the firm.^98^ As already mentioned, these figures were attributable not to the promotional activities of the Marketing Department, but rather to the drug itself, and the interest it generated among researchers both inside and outside the company.^99^ An important milestone in tamoxifen’s tortuous route from quasi-orphan drug to commercial success was therefore the detailed and convincing case for the use of tamoxifen in breast cancer, which John Patterson, a member of ICI’s Clinical Research Department (formerly of the Medical Department), prepared and put to the FDA’s Oncological Drugs Advisory Committee in 1976, leading to its approval for the U.S. market in 1977.^100^[Other P-679]

Meanwhile, Weston Hurst had been succeeded by Dr. Steve B. Carter on the Virus and Cancer project, and from then on the project reflected Carter’s personal approach to the study of cancer. It was titled Cell Division and Growth, and was derived from his fundamental work on cell mobility, which had been published in a letter to *Nature* in 1965.^101^ However, no drug was to come out of this program, and when Steve Carter left the company, taking early retirement in 1977, he was not replaced, and the project on cell growth was terminated. Thus in 1980, when tamoxifen was bringing in sizeable profits for the company and Zoladex (for prostate cancer) was in the pipeline, ICI had no longer a cancer research program, a situation that lasted until recently, when Alderley Park became the Global Lead Centre for the company’s cancer research.^102^

As to tamoxifen, it continued its complex trajectory from bench to bedside, changing the market for anticancer drugs in the process. Indeed, it played a key part in three developments that helped to shape the future of breast cancer therapy. The first was the isolation of the estrogen receptor. This led to heightened scientific interest in drugs that could bind to the receptor, like tamoxifen, and therefore became useful research tools in investigations of hormone-dependent tumors.^103^ The second was the development of adjuvant therapy for breast cancer. Until then, adjuvant therapy had consisted either in chemotherapy or in major endocrine ablation after curative surgery. A large-scale study undertaken under the patronage of the Nolvadex Adjuvant Trial Organisation (NATO) changed the modalities of adjuvant therapy for breast cancer while helping to establish tamoxifen in the treatment of the early stages of the disease.^104^ It was in the context of these adjuvant trials that evidence also emerged of the drug’s potential to *prevent* the recurrence of breast cancer in women at high risk (i.e., who had already had cancer in one breast), thus making tamoxifen the first preventive for any cancer and helping to establish the [Other P-680] broader principles of chemoprevention, and extending the market for tamoxifen and similar drugs further still.^105^

Like so many other anticancer drugs, tamoxifen, one of ICI’s most successful remedies, therefore began life as an “orphan” drug. It survived at first only because it had a committed drug champion in the person of Arthur Walpole, and later because of growing interest on the part of the wider scientific and medical communities. Nevertheless, the example of tamoxifen suggests that, from its experience with the beta-blockers, ICI had learned new ways of innovating with the use of (1) the Development Programme to move its lead drug on from the bench to the bedside and (2) the FDA in order to target the American market for medicines, and that it applied this lesson to the cancer field. ICI also benefited from its preexisting ties to DuPont, its link with the Anglo-American World War II collaborative research programs, and its long-term focus on the American market, which helped to shape its pharmaceutical R&D program after the war. One may therefore expect to find major differences as well as similarities between the trajectories of ICI and its French counterpart, Rhône-Poulenc, in terms of their marketing strategies in the cancer field.

## Rhône-Poulenc, Chlorpromazine, and the American Medical Market

Rhône-Poulenc was formed in 1928 from the merger between the pharmaceutical firm Poulenc-Frères and the chemical company la Société des Usines Chimiques du Rhône (SUCR). At the same time, a selling company was founded, named Spécia (derived from the word “spécialités,” i.e., ethical preparations), with the purpose of bringing together the pharmaceutical activities of the new group,^106^ and the British company May & Baker became its wholly owned subsidiary, thereby giving it access to new [Other P-681] markets in the British Empire.^107^ From the beginning, Poulenc-Frères had been one of the most innovative pharmaceutical companies in France. It built one of the first industrial research laboratories in the country, and embarked on the development of synthetic drugs modeled on the products of the German dyestuffs industry. Many of these drugs were developed by the pharmacist Ernest Fourneau, who continued his collaboration with Poulenc after leaving the company in 1911 to direct the new Therapeutic Chemistry Laboratory at the Pasteur Institute in Paris, and again after the merger between Poulenc and SUCR in 1928.^108^ The relationship between the Therapeutic Chemistry Laboratory and Rhône-Poulenc was to play an important role in the development of a modern French pharmaceutical industry, resulting in the discovery of sulphanilamide in 1936, and later in the development of synthetic antihistamines, curare analogues, and the psychotropic drug chlorpromazine.^109^ With this drug, the first of its kind, Rhône-Poulenc successfully targeted the American market. I therefore say a little about its history here, before comparing it to its anticancer drugs.

Like many of ICI’s drugs, chlorpromazine was the product of different wartime research projects, the first on synthetic antihistamines, the second on antimalarials. Both projects emerged from the study of compounds with which we are now quite familiar: phenylethylene and its analogues. But unlike ICI, Rhône-Poulenc carried out this research under the constraints of the German occupation, which resulted in the French group’s relative isolation and exclusion from the Allies’ collaborative research programs. Such constraints and isolation did not stifle innovation however. On the contrary, they may even have stimulated it, as illustrated by the history of the synthetic antihistamines.^110^ The outgrowth of a doctoral project carried out in the 1930s in Fourneau’s laboratory under the supervision of Daniel Bovet, Rhône-Poulenc’s antihistamine project [Other P-682] led to what was arguably the group’s first truly innovative medicine: a phenylethylenediamine derivative, which was found to be useful in the treatment of allergies, and was launched under the name of Antergan by Spécia in 1942.^111^ However, Antergan’s inventor, Bernard Halpern, who was Jewish, soon had to leave France for Switzerland to avoid the convoys of racial deportees that followed Germany’s occupation of the Southern Zone.^112^ His research was therefore pursued by Rhône-Poulenc in collaboration with Daniel Bovet, who returned to the antihistamine work with the help of Halpern’s assistant, France Walthert. This led in 1944 to another phenylethylenediamine, marketed as Néo-Antergan.^113^ Then, as the conflict drew to a close, work began on a new series of derivatives, the phenothiazine amines, which had been synthesized by the Rhône-Poulenc chemist Paul Charpentier in search of antimalarial drugs.^114^ Unaware that an American team had also synthesized these compounds, but found them worthless as antimalarials, the French team had pressed on with their research and made compound 3276 RP (the “RP” standing for Rhône-Poulenc). Although shelved at the time, 3276 RP would later serve as a building block for the chlorpromazine molecule.^115^ In the pharmacological screens used to detect antihistamine activity, Bernard Halpern, who by then had been able to return from Switzerland, selected the most active and least toxic of the phenothiazine series: promethazine, which was marketed under the name Phénergan, and became Rhône-Poulenc’s third and most successful antihistamine drug.

Before long, the unique properties of the synthetic antihistamines started attracting the attention of researchers and clinicians. One of these was the French naval surgeon Henri Laborit, who found that the central (sedative and hypnotic) effects of Phénergan were helpful in surgery, and used it as part of a cocktail of drugs to counteract the effects of shock in his patients. In 1948 Laborit gave a talk in front of Rhône-Poulenc’s therapeutic research team, and after his talk, the team returned to the phenothiazines prepared earlier by Charpentier, looking for central [Other P-683] activity in order to increase it, rather than suppress it as they had done previously.^116^ Using 3276 RP as his starting point, in December 1950 Charpentier synthesized 4560 RP, later named chlorpromazine, which underwent pharmacological tests and was released for clinical investigation in France in 1951.^117^

Seeing an analogy between shock and mental disorders, Laborit introduced the drug, now known as Largactil, to his colleagues at the psychiatric hospital Sainte Anne in Paris.^118^ There, Jean Delay and Pierre Deniker established what would become the standard method of clinical application of Largactil, in isolation rather than as part of a cocktail, a method that would facilitate its adoption by psychiatrists across the world.^119^ In association with this method, chlorpromazine was exported, first to Britain, via Rhône-Poulenc’s subsidiary May & Baker, and later to the United States, where it was relatively quickly taken up, despite the strength of its psychoanalytic tradition, thanks to a well-designed marketing campaign, and to the active collaboration of clinicians such as Laborit and Deniker.^120^ The history of the worldwide adoption of the drug is, according to Judith Swazey, a reflection of the drug’s success.^121^ It is also a reflection of the firm’s success at entering the American market for drugs, which I turn to now.

Rhône-Poulenc had begun targeting the American market with its antihistamine drugs. It therefore offered chlorpromazine to the same companies that sold the antihistamines on its behalf in the United States, but which turned it down. Then, in April 1952, J. Monnet, Rhône-Poulenc’s director for agreements and patents, wrote to Francis Boyer, president of SmithKline & French (SK&F), offering the American pharmaceutical firm a licensing agreement to manufacture and market chlorpromazine in the United States. With his letter, he enclosed an article by Laborit and an internal note describing the pharmacodynamic properties of the drug.^122^ Boyer, who for two years had been “wooing” Rhône-Poulenc in [Other P-684] an effort to establish an agreement to exchange compounds and information with the French group, was most interested in what he read. He therefore requested five hundred grams of the compound so that his company could carry out initial laboratory and clinical tests. At the time SK&F had been seeking to develop its own nonbarbiturate sedative or hypnotic drugs. Therefore it already had a pharmacological screen with which to test for activity, as well as a product that could be sent to Rhône-Poulenc in exchange for chlorpromazine (but which eventually turned out to be ineffective in humans).^123^ At this stage SK&F was mainly interested in chlorpromazine as a potentiator of anesthesia, although the treatment of acute manias was also considered, and it began laboratory tests and small-scale clinical trials to reach a final decision on whether or not to market the drug. In the process SK&F found that, unlike their French colleagues, American surgeons had little enthusiasm for using chlorpromazine in anesthesia. Instead, SK&F therefore envisaged four different areas of clinical trial: nausea and vomiting, general sedation, antipruritic activity (to relieve the itching associated with Hodgkin’s disease for example), and psychiatric conditions, although at first trials in such conditions remained limited.^124^ However, by 1953 evidence had begun to accumulate in France on the usefulness of chlorpromazine in psychiatry, and SK&F identified a number of likely clinical centers for psychiatric studies in the United States. The American company also began work on a New Drug Application (NDA) and stepped up its clinical trial program after obtaining market authorization from the FDA for chlopromazine. Within eight months of it being launched under the name Thorazine, around eight million patients were taking the drug, and within a year of its introduction, it had boosted SK&F’s sales by one-third.

SK&F handled all the crises that faced chlorpromazine on its American journey from bench to bedside, from the initial rejection of Rhône-Poulenc’s American patent application to the series of side effects observed during the clinical trials performed in 1953, which could potentially have threatened the NDA, but which SK&F eventually obtained on behalf of Rhône-Poulenc in March 1954. By identifying all “the needs and options that CPZ [chlorpromazine] helped to create for the operation of mental hospitals and for the care of a new and growing non-hospitalized patient population,”^125^ SK&F also played a key role in introducing chlorpromazine into American psychiatry, which would eventually become one of its most lucrative markets. Having overcome these obstacles and developed these [Other P-685] options, SK&F reaped the rich rewards of its efforts with Thorazine, as did Rhône-Poulenc. Thanks to SK&F, the French group had succeeded in surmounting the difficulties that faced European drug companies attempting to enter the American market in the 1950s, “such as the dollar shortage, the 1938 Food and Drug Act regulations, and the scepticism of American physicians toward European scientific data and clinical work.”^126^

Rhône-Poulenc’s success with chlorpromazine inspired the development of its subsequent psychotropic drugs, and helped to lay the foundations of the group’s expansion in the 1950s and beyond, transforming the practice of psychiatry and giving rise to the new field of psychopharmacology in the process.^127^ However, its success had relied upon an American firm, SK&F, and was not easily transferrable to other therapeutic domains, as we shall see with its anticancer drugs.

## Rhône-Poulenc and Cancer Research

Until World War II, Rhône-Poulenc’s competences in pharmaceuticals had resided essentially in the field of synthetic organic chemistry. However, during the war, the group became involved in the development of penicillin, a development that marked its entry into the antibiotics industry and laid the foundations of its cancer chemotherapy program. It was to have a major impact on Rhône-Poulenc’s R&D activities, which grew along two principal axes after the war: (1) synthetic drugs and (2) antibiotics.^128^ In terms of marketing strategy, the French group displayed distinctive styles in the two different (essentially traditional, product-based) areas. Whereas in the field of synthetic drugs Rhône-Poulenc showed itself to be confident, succeeding as we have seen with chlorpromazine thanks to the combined use of consultants and licensees, in the antibiotics field, and by extension that of cancer chemotherapy, the French firm remained diffident toward, and only remotely engaged with, American academics, companies, and the U.S. market.

Although Rhône-Poulenc had gained a foothold in the antibiotics field independently thanks to its early involvement with penicillin, its wider antibiotic program originated in the competences the French group acquired under license from the American company Merck, with whom [Other P-686] the French firm entered into a contract in August 1945 to manufacture penicillin by deep fermentation methods. By 1947, this had become a two-way contract, through which Merck gained access to Rhône-Poulenc’s knowledge and know-how on synthetic antihistamines and Flaxedil, a curare-like muscle relaxant, in exchange for which the French group also obtained information about cortisone and vitamin B12, as well as a license for the production of streptomycin by submerged culture.^129^ Building on this knowledge and know-how, Rhône-Poulenc was able to develop spiramycin, pristinamycin, and the semisynthetic antibiotic metronidazole (still used today for the treatment of parasitic infections, including trichomonas). In the 1950s, antibiotics had been among the substances screened by the CCNSC.^130^ One of the first antibiotics to show activity against cancer in these screens was Merck’s anthracyclin antibiotic, Actinomycin D, which belonged to the streptomyces family of antibiotics.^131^ The discovery of the cytotoxic properties of Actinomycin D, marketed by Merck as Dactinomycin, prompted Rhône-Poulenc to initiate its own cancer research program in 1956.^132^ This led to the identification of the cytotoxic properties of its own anthracyclin antibiotic, rufochromomycin, which had been isolated earlier, in 1952. The Rhône-Poulenc laboratories involved in this cancer research program included newly established laboratories, built for the purpose, such as the Cancerology laboratories, as well as older ones: Biochemistry and Fermentation, and Organic Chemistry (for the discovery of new compounds); Pharmacology and Toxicology, and Analytical Chemistry (for further investigation of new compounds).^133^

In 1963, Rhône-Poulenc’s cancer research program was further strengthened by the acquisition of the Roger Bellon Laboratories, a firm founded in 1922 with technical expertise in microbiology, and which after World War II began producing antibiotics.^134^ This acquisition led to the group’s first major anticancer drug, daunorubicin (Cérubidine), which [Other P-687] like its predecessors, spiramycin, pristinamycin, and rufochromomycin, had been isolated from a strain of streptomyces, in 1962, and is still part of the medical armamentarium against cancer today. Daunorubicin was found to be active against leukemia in the firm’s pharmacological cancer screens, and was tested in the clinic by the physicians Jean Bernard and Claude Jacquillat at the Saint-Louis Hospital in Paris, in 1964. It was followed soon after by rubidazon, a semisynthetic derivative of daunorubicin which was obtained in 1968, and was also tested in leukemia, in 1971.

By then it was clear that Rhône-Poulenc had two not only viable but also potentially profitable anticancer drugs, for which the American market was an important target. Therefore, the Yves Laboratories were contracted to sell the products in the United States and help obtain market authorization from the FDA, a practice that Rhône-Poulenc had adopted before and that many other firms commonly adopted in the United States. However, Yves Laboratories were not having much success, and the NDA to the FDA for daunorubicin was not going well: not only did the samples of daunorubicin turn out to be contaminated by penicillin, but the application needed to be rewritten to conform with the FDA’s requirements.^135^ Moreover, daunorubicin already had a serious competitor in adriamycin (a Farmitalia product), which had been championed by the influential Stephen K. Carter, deputy director for cancer therapy evaluation, Division of Cancer Treatment at the Department of Health, Education and Welfare in Bethesda, and as a result was becoming well established in the United States, even though Rhône-Poulenc thought it to be inferior to its own product.^136^ The French group had long been sending its compounds for screening to the CCNSC, and was continuing to do so well into the 1970s, although by then it had developed considerable screening capabilities of its own. Nevertheless, from the daunorubicin/adriamycin episode it concluded that in the cancer field it “should propose its most interesting compounds for testing to the NCI very early on,” as any delays in doing so would only accentuate the problems in obtaining market authorization from the FDA.^137^

Then in 1974, Rhône-Poulenc struck lucky: a proposal to prepare an Investigational New Drug (IND) application for rubidazon independently of the NIH, and to devise phase I trials, was received from Robert S. Benjamin, assistant professor of medicine and pharmacology at the University of Texas System Cancer Center, MD Anderson Hospital and Tumor Institute, [Other P-688] in Houston.^138^ This offer of collaboration, which was the result of personal contacts established between René Maral (responsible for the Cancerology laboratories at Rhône-Poulenc) and Benjamin, enabled the French group to obtain for the first time in 1975 an IND without any assistance from Yves, or any other licensee.^139^ Benjamin was promptly invited to become a consultant for the company, which he accepted in 1977.

**Figure 3:**
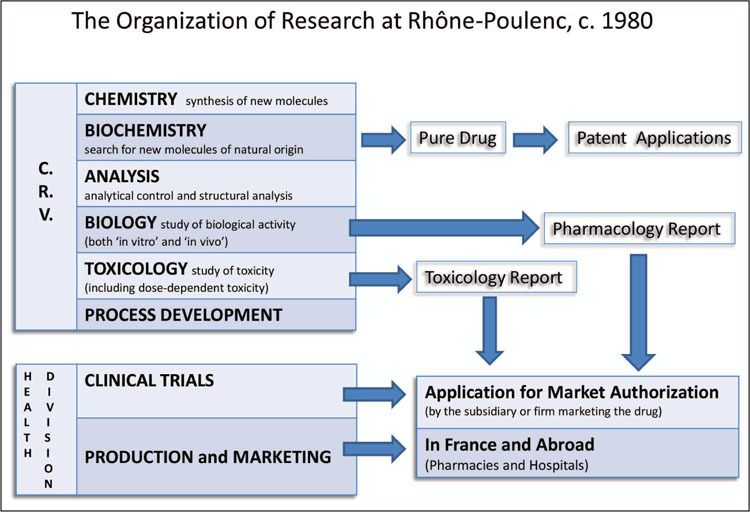
Organization of Research at Rhône-Poulenc, c. 1980. Source : Schema simplifié de l’organisation des recherches, Sanofi-Aventis Historical Photograph Collection.

Having abandoned the idea of using Yves, Rhône-Poulenc’s approach to the American market for anticancer drugs therefore privileged collaborative relations with clinical researchers in order to influence medical opinion and—eventually—smooth the path to obtaining market authorization from the FDA, rather than targeting the FDA directly and systematically, as ICI did with their beta-blockers and then with tamoxifen. This dependence on clinicians was situated in the gap between the research stage, which was the responsibility of the company’s main research department (Centre de Recherche de Vitry), and the development stage (including clinical trials, manufacturing, and marketing), overseen by the firm’s Health Division (Division Santé; see Figure 3).

[Other P-689]

Rhône-Poulenc’s relations with clinicians read like a who’s who of cancer research, to begin with especially in France,^140^ and from the 1970s extending this network to the United States in an attempt to find intermediaries with the FDA and gain access to the American market. These relations were developed first through informal links, via correspondence, publications, membership of expert networks, and attendance at conferences, before they were formalized into consultancies. They therefore existed in a variety forms, which evolved over time: from payments of the salaries of technicians working in hospital laboratories to honoraria given to consultants for work on specific projects or for more general advice and participation in cooperative groups for the development of cancer therapies (see Table 2).

However, even the best of relations did not prevent missed opportunities, as in the 1970s when Rhône-Poulenc turned down the French chemist Pierre Potier’s offer of the semisynthetic Vinca alkaloid Navelbine (vinorelbine), which instead was developed by the Pierre Fabre Laboratories and was approved by the FDA for use in the American market in 1994.^141^

In contrast to ICI, Rhône-Poulenc’s approach to marketing in the cancer field appeared at first to be largely ad hoc and intuitive, that is, the antithesis of “scientific marketing” in the scientific marketing era. This was in part due to the fact that the French market had long been protected by the French government through various measures such as import controls, but also to French industry’s technical and scientific dependency on American firms, especially in relation to antibiotics, upon which pharmaceutical expansion was largely based after World War II. However, all this was to change in the 1980s, when a major crisis led Rhône-Poulenc to being nationalized. Between 1982 and 1993, when it was under government control, it sold off several of its businesses in order to focus on its core capabilities in health, fine organics, and the biological industries, and to make its strategy more coherent. It also developed a new international policy, abandoning its earlier practice of licensing the patent rights for its products to companies abroad, instead building business partnerships with local manufacturers, which, as we saw with the example of chlorpromazine, it had already started to do in the pharmaceutical field.^142^ Finally, under the leadership of Igor Landau, a former McKinsey [Other P-690] consultant who joined Rhône-Poulenc in 1975 and later became head of the Health Division, the firm turned to American management consultants such as A. D. Little to carry out systematic surveys and quantitative analyses of potential markets, including the market for anticancer drugs.^143^ Unsurprisingly therefore, when Potier turned to the company again later with a proposal for a collaborative agreement to explore the chemistry of taxoids and seek new and patentable anticancer drugs, Rhône-Poulenc did not repeat the mistake it had previously made with Navelbine. The outcome of their collaborative arrangement was taxotère, which received FDA clearance for launch in the United States in 1996, and would become Rhône-Poulenc’s most successful anticancer drug.^144^

## Concluding Remarks

In this article, I have compared and contrasted ICI’s approach to the American market for medicines with Rhône-Poulenc’s, which appeared less well organized, confident, and coherent, depending on the therapeutic area that was being targeted: psychiatry or cancer. Whereas ICI’s R&D and marketing strategies were consistent with each other and aligned themselves on the requirements of the FDA, which was recognized early as the gatekeeper to the American market, Rhône-Poulenc’s R&D activities remained largely separate from each other as well as from its marketing strategy, the former focusing on clinical researchers as opinion leaders, the latter relying on licensees to market its drugs in the United States. The differences between the two firms were in part attributable to their different historical trajectories.^145^ While ICI benefited from its “special relationship” with DuPont and its involvement in Anglo-American collaborative research programs during World War II, Rhône-Poulenc suffered from relative isolation under German Occupation, and after the war relied heavily upon American know-how, particularly for its antibiotics, and on American companies to make and market its drugs in the United States.[Other P-691]

**Table 2:** Rhône-Poulenc’s Who’s Who Network in Cancer Research

Persons/institutions	Location/brief description	Dates
Dr. Pierre Jollès Biological Chemistry laboratory of the Science Faculty in Paris From 1966 researcher at the Institute of Cancerology and Immunogenetics (ICIG)	Brother of George Jollès—chemical engineer at Rhône-Poulenc	1950s–1960s
Prof. Georges Mathé Saint-Louis Hospital, then from 1964 ICIG	Founder and first director of ICIG, Paul-Brousse Hospital, Villejuif, Paris	1950s–1960s
Prof. Jean Bernard Hôpital Hérold, and from 1957 Saint-Louis Hospital	Director of Hayem Centre for Heamatological Research at the Saint-Louis Hospital from 1960	1950s–1960s
Prof. Claude Jacquillat	Head of Oncology Department at the Pitié-Salpêtrière Hospital	1950s–1960s
Fondation Bergonié	Cancer Research and Treatment Centre, Bordeaux	
Institut Gustave Roussy	Cancer Research and Treatment Centre, Villejuif, Paris	1980s–
Centre International de la Recherche sur le Cancer (opened in 1972)	Lyon	1970s–1980s
Institut de Chimie des Susbtances Naturelles Prof. Pierre Potier	Gif-sur-Yvette, near Paris	1970s–1980s
Prof. Robert S. Benjamin University of Texas System Cancer Center, MD Anderson Hospital and Tumor Institute, Houston	Assistant professor of medicine and pharmacology, Houston, Tex.	1970s–
Dr. F. J. C. Roe Royal Marsden Hospital	Chelsea, London	1970s
Dr. André Trouet, Laboratory of Physiological Chemistry, Catholic University of Louvain	Researcher in Prof. Christian de Duve’s Department (founder of the International Institute of Cellular and Molecular Pathology), Louvain, Belgium	1970s–
Laboratory of Molecular Biophysics Dr. C. Aubel-Sadron	CNRS, Orléans	1980s–

[Other P-693]

However, in the cancer field, which had many specificities, especially the pivotal role played by hospital clinicians in establishing the modalities of cancer chemotherapy, and was problematic for all drug companies until chemotherapy became accepted as a therapeutic approach to the treatment of cancer, Rhône-Poulenc’s strategy of targeting key opinion leaders does not seem so ill adapted. Moreover, Rhône-Poulenc showed an ability to learn from its mistakes as well as successes, modifying its strategy in the light of previous experience.

Just as ICI had learned new ways of innovating from its experience with the beta-blockers and applied this lesson to the cancer field, Rhône-Poulenc’s success with chlorpromazine led the French group to try to repeat the experience with its anticancer drugs. When it failed to attract a company of the caliber of SK&F, it adopted the same approach that had succeeded at home, privileging collaborative relations with clinicians in order to gain a foothold in the American market (see Table 2). Rhône-Poulenc’s approach is all the more understandable in that it was attempting to enter this market with antibiotics, which not only were the product of its dependency on American know-how, but resembled several other drugs in a market fast becoming overcrowded by competitors. In contrast, tamoxifen, like chlorpromazine, but unlike daunorubicin and rubidazon, may have had a better chance of succeeding, and therefore fashioning its own medical market, because it was the first of its kind.

Hence the two companies’ different approaches to the American market for anticancer drugs appear quite rational, if not always “scientific” in the context of “scientific marketing,” as they were the product of the different historical origins and technical nature of their cancer research programs. For the French group, like its British counterpart, “the scale of American cancer research was so vast that it raised important questions about how to fit into this research world.”^146^ In answer to such questions, even in the age of scientific marketing, “intuitive” marketing therefore offered (and still offers) helpful solutions. This conclusion confirms and reinforces the point made by historians of early modern medicine, that “there are other stories to be told and other relationships than that of buyer and seller to explore.”^147^ For even in the modern medical marketplace, there are relationships and activities that “do not fit neatly into consumerism,”^148^ for instance the relations of reciprocal patronage between clinicians and companies, or between R&D and marketing.[Other P-694]

By carrying out a multilayered comparison of two foreign drug companies that targeted the American market for medicines, helping to construct it through their R&D and marketing activities, and were transformed in the process, this article has described the persistent differences between firms, their drugs, and the diseases they aim to treat. Why are such differences worth highlighting? By studying how they shaped modern medical practice, it may well be possible to influence and perhaps modify some of its more worrying trends, in particular an overreliance on drugs to prevent as well as treat diseases, and a growing disillusion with modern medicine.

Addressing the first concern, this article has shown through the example of ICI that since World War II interactions between companies and regulatory agencies have grown in significance, while Rhône-Poulenc illustrates the enduring relations between companies and clinicians and their lasting role in shaping the medical market. Both cases therefore suggest a couple of pressure points for intervention in pharmaceutical regulation and health policy: the relationship of private companies with regulatory agencies on the one hand, and with clinical medicine on the other.

In response to the second concern, that is, the mounting disillusion with modern medicine, the lengthy and often painstaking accumulation of knowledge and know-how that underpins innovative medicines not only provides hope for the future, but also emphasizes the need for incentives to encourage sustained investment in areas of public health significance. More specifically in relation to cancer, Rhône-Poulenc and ICI began their formal cancer research programs in the 1950s, yet their first successful anticancer drugs did not truly emerge until the 1980s, the former with taxotère, which built upon knowledge in oncology and know-how in semisynthetic antibiotics, the latter with tamoxifen, which from its beginnings as a contraceptive pill was “reinvented” first as a chemo-therapeutic, and then as a chemo-preventive anticancer drug.^149^ In between the 1950s and 1980s, Rhône-Poulenc and ICI had to acquire new scientific knowledge and technical expertise, as well as learn how to engage with a foreign market: the American market for medicines. This engagement also involved the accumulation of new knowledge and proficiency, albeit of a different kind: in dealing with regulatory agencies, in R&D organization, as well as in marketing. To stimulate such a lengthy and complex learning process in fields other than cancer, political willpower and leadership on the part of public health authorities may be required.[Other P-695]

Last but not least, this article has underlined the importance of considering different drug and disease categories, not only because of the intimate relationship that has developed between them, but because they are at the very heart of modern medicine, thereby grounding studies such as this in the physical and experiential nature of medicinal substances and the conditions they treat.[Other P-696]

## Footnotes

1

GreeneJeremy A. , Prescribing by Numbers: Drugs and the Definition of Disease (Baltimore: Johns Hopkins University Press, 2007), xi.

2 Ibid., 3–4.

3 Ibid., 3.

4

SismondoSergio , “Linking Research and Marketing: A Pharmaceutical Innovation,” in Perspectives on Twentieth-Century Pharmaceuticals, ed. QuirkeVivianeSlinnJudy (Oxford: Peter Lang, 2010), 241–56.

5
See, e.g., GaudillièreJean-Paul , Inventer la biomédecine: la france, l‘amérique et la production des savoirs du vivant (Paris: La Découverte, 2002); KrigeJohn , American Hegemony and the Postwar Reconstruction of Science in Europe (Cambridge, Mass.: MIT Press, 2006 QuirkeVivianeGaudillièreJean-Paul , “The Era of Biomedicine: Science, Medicine and Public Health in Britain and France after the Second World War,” Med. Hist. 52 (2008): 441–52; QuirkeViviane , “Anglo-American Relations and the Co-production of American ‘Hegemony’ in Pharmaceuticals,” in American Firms in Europe, 1880–1900:Strategy, Identity, Perception and Performance, ed. BoninHubertde GoeyFerry (Geneva: Droz, 2009), 363–84; more specifically on pharmaceutical regulation, see CarpenterDaniel , Reputation and Power: Organizational Image and Pharmaceutical Regulation at the FDA (Princeton: Princeton University Press, 2010), chap. 11.

6
The radical agenda in the social construction of science, technology, and medicine has long argued that a better understanding of how change occurs has implications for both politics and policy. More specifically in relation to technology, see PinchTrevor , “The Social Construction of Technology: A Review,” in Technological Change: Methods and Themes in the History of Technology, ed. FoxRoberty (Amsterdam: Harwood, 1996), 17–35, 35; in relation to health policy, see BerridgeVirginia , “History Matters? History’s Role in Health Policy Making,” Med. Hist. 52 (2008): 311–26.

7
“Therapeutic Revolution” was coined by the American historian of medicine RosenbergCharles E. in his classical essay titled “The Therapeutic Revolution: Medicine, Meaning and Social Change in Nineteenth-Century America,” Perspect. Biol. Med. 20, no. 4 (1977): 485–506. Since then, it has often been used to refer to the pharmacological revolution of the mid-twentieth century, i.e., the explosion in the number of drug treatments for a wide variety of ailments, and more recently the behavioral turn in certain fields of medicine, in particular those relating to mental health. See BasmajianJ. V. , “The Third Therapeutic Revolution: Behavioral Medicine,” Appl. Psychophysiology Biofeedback 24, no. 2 (1999): 107–16.

8
The classic skeptical view of medicine’s contribution to health and welfare is Tom McKeown’s The Role of Medicine: Dream, Mirage or Nemesis? (Oxford: Blackwell, 1979). See also Le FanuJames , The Rise and Fall of Modern Medicine (London: Abacus, 2000).

9

Carpenter , Reputation and Power (n. 5).

10
For an overview of the history of the pharmaceutical industry, see, e.g., GoodmanJordan , “Pharmaceutical Industry,” in Medicine in the Twentieth Century, ed. CooterRogerPickstoneJohn (Amsterdam: Kluwer, 2000), 141–54.

11
For example, SantesmasesMaría Jesús , “Distributing Penicillin: The Clinic, the Hero and Industrial Production in Spain, 1943–1952” in QuirkeSlinn , Perspectives on Twentieth-Century Pharmaceuticals (n. 4), 91–117; QuirkeViviane , “Making British Cortisone: Glaxo and the Development of Corticosteroids in Britain in the 1950s and 1960s,” Stud. Hist. Philos. Biol. Biomed. Sci. 36 (2005): 645–74.10.1016/j.shpsc.2005.09.00116337555

12

RasmussenNicolas , “The Moral Economy of the Drug Company-Medical Scientist collaboration in Interwar America,” Soc. Stud. Sci. 34 (2004): 161–85; TobbellDominique , “Allied Against Reform: Pharmaceutical Industry-Academic Physician Relations in the United States, 1945–1970,” Bull. Hist. Med. 82 (2008): 878–912. According to Sismondo and others, the relationship is so close as to influence the very content of medical science. SismondoSergio , “Ghost Management: How Much of the Medical Literature Is Shaped Behind the Scenes by the Pharmaceutical Industry?,” PLOS Med. 4, no. 9 (2007): e286; MathesonA. , “Corporate Science and the Husbandry of Scientific and Medical Knowledge by the Pharmaceutical Industry,” Biosocieties 3 (2008): 355–82.

13

HealyDavid , Let Them Eat Prozac: The Unhealthy Relationship between the Pharmaceutical Industry and Depression (New York: New York University Press, 2004); MedawarC.HardonA. , Medicines Out of Control? Antidepressants and the Conspiracy of Goodwill (Amsterdam: Aksant, 2004).

14
For example, CritserG. , Generation Rx: How Prescription Drugs Are Altering American Lives, Minds and Bodies (New York: Houghton Mifflin, 2005); ToneAndrea , The Age of Anxiety: A History of America’s Turbulent Affair with Tranquilizers (New York: Basic Books, 2009); on the link between pharmaceutical marketing and medical research and practice in diabetes and cardiovascular diseases, see Greene , Prescribing by Numbers (n. 1).

15
For a definition of innovative medicines, see MorganS.LopertR.GreysonD. , “Toward a Definition of Pharmaceutical Innovation,” Open Med. 2 (2008): 4–7. On the relationship between R&D and marketing, see, BeckerM. C.LillemarkM. , “Marketing/R&D Integration in the Pharmaceutical Industry,” Res. Policy 35 (2006): 105–20; see also online debate with an article in *Science Daily*, “Big Pharma Spends More on Advertising Than Research and Development, Study Finds,” Sci. Daily, 1 7, 2008, http://www.science-daily.com/releases/2008/01/080105140107.htm; for a counterargument, see LoweDerek , “Drug Industry Spending: R&D vs. Marketing,” Seeking Alpha, 7 9, 2009, http://seekingalpha.com/article/147881-drug-industry-spending-r-d-vs-marketing; the debate has even made it onto YouTube with Marcia Angell, “The Truth about Drug Companies: R&D, Marketing,” http://www.youtube.com/watch?v=kWLQpnRlt5A (accessed October 30, 2013), based on her book *The Truth about Drug Companies*.

16

SlinnJudy , “‘A Cascade of Medicines’: The Marketing and Consumption of Prescription Drugs in the UK, 1948–2000,” in From Physick to Pharmacology: Five Hundred Years of British Drug Retailing, ed. CurthLouise Hill (Aldershot: Ashgate, 2006), 143–69, quotation on 159. Quoted from Sainsbury Committee Report, National Archives, Kew, Cmnd 3410, 1967.

17

CorstjensMark , Marketing Strategy in the Pharmaceutical Industry (London: Chapman & Hall, 1991), chap. 6, esp. 128.

18 Ibid., chap. 7.

19
Ibid., 156. For more on the impact of thalidomide on pharmaceutical R&D, see QuirkeViviane , “Thalidomide, Drug Safety Regulation and the British Pharmaceutical Industry: The Case of Imperial Chemical Industries,” in Ways of Regulating Drugs in the 19th and 20th Centuries, ed. GaudillièreJean-PaulHessVolker (Basingstoke: Palgrave Macmillan, 2013), 151–80.

20

Corstjens , Marketing Strategy (n. 17), 155, 184.

21
See TadajewskiM.JonesD. G. Brian , eds., History of Marketing Thought (Thousand Oaks, Calif.: Sage, 2008). Although scientific marketing has a long history stretching back to F. W. Taylor and the birth of scientific management at the end of the nineteenth century, it took off in the period between the wars, influenced by publications such as Claude Hopkins’s 1923 volume *Scientific Advertising* Spurred by wartime developments in operations research and management science models targeting mass markets in the 1950s and 1960s, and relayed by American firms of management consultants, it then spread from the United States to Europe. For a history of scientific management, see, e.g., MerkleJ. A. , Management and Ideology: The Legacy of the International Scientific Management Movement (Berkeley: University of California Press, 1980). More specifically on marketing science/scientific marketing, see the special issue of Journal of Marketing (Fall 1983).

22

Corstjens , Marketing Strategy (n. 17), 137.

23 Ibid., 166.

24

KeatingPeterCambrosioAlberto , “Cancer Clinical Trials: The Emergence and Development of a New Style of Practice,” in “Cancer in the Twentieth Century,” ed. CantorDavid , Bull. Hist. Med. 81 (2007): 197–223.10.1353/bhm.2007.000317369668

25

Corstjens , Marketing Strategy (n. 17), 133.

26

PinellPatrice , Naissance d’un fléau (Paris: Eds Métaillé, 1992); Pinell , “Cancer,” in Companion to Medicine in the Twentieth Century, ed. CooterRogerPickstoneJohn (London: Routledge, 2003), 671–86. Pinell points out that the perception of cancer as a “scourge” predated any epidemiological evidence of it being on the increase.

27

Corstjens , Marketing Strategy (n. 17), 144, figure 6.8.

28
See box 2, “The Growth of Chemotherapy as an Industry,” in ChabnerB. A.RobertsT. G.Jr. , “Chemotherapy and the War on Cancer,” Nature Rev. Cancer 5 (1 2005): 65–72, 70 See also GarattiniS.BerteleV. , “Efficacy, Safety, and Cost of New AntiCancer Drugs,” Brit. Med. J. 325 (2002): 269–71.

29

Corstjens , Marketing Strategy (n. 17), 140–45. Cancer is not the only therapeutic area where there is a close relationship between drugs and the market for a particular disease. On drugs and cardiovascular diseases, see VosRein , Drugs Looking for Diseases: Innovative Drug Research and the Development of the BetaBlockers and the Calcium Antagonists (Dordrecht: Kluwer, 1991).

30

BudRobert , “Strategy in American Cancer Research after World War II: A Case Study,” Soc. Stud. Sci. 8 (1978): 425–59; DeVitaV. T.Jr.ChuE. , “A History of Cancer Chemotherapy,” Cancer Res. 68 (2008): 8643–53.10.1177/03063127780080040211610432

31

WeatherallMiles , In Search of a Cure: A History of Pharmaceutical Discovery (Oxford: Oxford University Press, 1990), chap. 11; LöwyIlanaGaudillièreJean-Paul , “Disciplining Cancer: Mice and the Practice of Genetic Purity,” in The Invisible Industrialist: Manufactures and the Production of Scientific Knowledge, ed. GaudillièreJean-PaulLöwyIlana (London: Macmillan, 1998), 209–49; DeVitaChu , “History of Cancer Chemotherapy” (n. 30).

32

RaviñaE. , The Evolution of Drug Discovery: From Traditional Medicines to Modern Drugs (Weinheim: Wiley-VCH Verlag & Co. KGaA, 2011), 95–96.

33

SneaderWalter , Drug Discovery: A History (Chichester: Wiley, 2005), 440.

34

Weatherall , In Search of a Cure (n. 31), chap. 11.

35 Alkylating agents are highly reactive chemical compounds that bond with various groups in nucleic acids and proteins and can be toxic to cancer cells.

36

Sneader , Drug Discovery (n. 33), 440–45.

37

LathropA.LoebL. , “On the Part Played by Internal Secretion in the Spontaneous Development of Tumors,” J. Cancer Res. 1 (1916): 1–19.12264098

38

LacassagneAntoine , “Apparition des Cancers de la Mamelle chez la Souris Mâle Soumise à des Injections de Folliculine,” Comptes Rendus de l’Académie des Sciences 195 (1932): 630; Lacassagne , “A Comparative Study of the Carcinogenic Action of Certain Oestrogenic Hormones,” Amer. J. Cancer 28 (1936): 735–40. Although the latter article had a significant impact on cancer research and treatment, interestingly, few histories of cancer chemotherapy mention Lacassagne’s contribution, perhaps because he lost interest in clinical practice and from 1935 confined himself to laboratory studies. ChamakBrigitte , Cent ans de recherches en cancérologie: le rôle d’Antoine Lacassagne (1884–1971) (Paris: Editions Glyphe, 2011), chap. 4, esp. 104–13. See also Chamak , “Un Scientifique pendant l’Occupation: le Cas d’Antoine Lacassagne,” Revue d’Histoire des Science 57 (2004): 101–33.

39

DoddsE. C.GoldbergL.LawsonW.RobinsonR. , “Synthetic Oestrogenic Compounds Related to Stilbene and Diphenylethane. Part I,” Proc. Roy. Soc,. B. 127 (1939): 140–67.10.1098/rspb.1953.000313027278

40

HugginsC.HodgesC. V. , “Studies on Prostatic Cancer: 1. The Effects of Castration, of Estrogen and of Androgen Injection on Serum Phosphatases in Metastatic Carcinoma of the Prostate,” Cancer Res. 1 (1941): 293–97.

41

NesbitR. M.BaumW. C. , “Endocrine Control of Prostatic Carcinoma,” J. Amer. Med. Assoc. 143 (1950): 1317–20.1542826110.1001/jama.1950.02910500019005

42

HaddowA.WatkinsonJ. M.PatersonE. , “Influence of Synthetic Oestrogens upon Advanced Malignant Disease,” Brit. Med. J. 2 (1944): 393–98.2078566010.1136/bmj.2.4368.393PMC2286289

43 The first notable successes with these hormones were achieved by William Dameshek and Sidney Farber’s teams at Harvard Medical School in Boston and by Joseph Burchenal’s at the Sloan Kettering Institute in New York.

44

WoodsD. D. , “The Relation of p-aminobenzoic Acid to the Mechanism of the Action of Sulphanilamide,” Brit. J. Exp. Pathol. 21 (1940): 74–90.

45

FildesPaul , “A Rational Approach to Research in Chemotherapy,” Lancet 235 (5 25, 1940): 955–57; Weatherall , In Search of a Cure (n. 31), 153–54, 222–23.

46

Raviña , Evolution of Drug Discovery (n. 32), 372–73.

47

AngierR. B. , “The Structure and Synthesis of the Liver L. Casei Factor,” Science 103 (1946): 667–69.20982775

48

RigalChristelle , “‘Neo-Clinicians, Clinical Trials, and the Reorganization of Medical Research in Paris Hospitals after the Second World War: The Trajectory of Jean Bernard,” in QuirkeGaudillière , “Era of Biomedicine” (n. 5).10.1017/s0025727300003008PMC257044418958252

49

Sneader , Drug Discovery (n. 33), 250–52. See also FanuLe , Rise and Fall (n. 8), chap. 10.

50

HitchingsG. H. , “Chemotherapy and Comparative Biochemistry,” G.H.A. Clowes Memorial Lecture, Cancer Res. 29 (1969): 1895–1903, here 1896.4982548

51 Ibid., 1895.

52

HitchingsG. H. , “Studies on Analogs of Purines and Pyrimidines,” Ann. N.Y. Acad. Sci. 52 (1950): 1318–35.1543314910.1111/j.1749-6632.1950.tb54032.x

53

Raviña , Evolution of Drug Discovery (n. 32), 374–76; Weatherall , In Search of a Cure (n. 31), 224–27.

54 See note 8.

55

Weatherall , In Search of a Cure (n. 31), 181.

56

Sneader , Drug Discovery (n. 33), 311. It was later found that these antibiotics act by intercalating with DNA, i.e., by reversible inclusion of a molecule or group between two DNA strands.

57

FanuLe , Rise and Fall (n. 8), 146–47; MacGregorA. B. , “The Search for a Chemical Cure for Cancer,” Med. Hist. 10 (1966): 374–86, 383.10.1017/s0025727300011480PMC10336425920367

58

Raviña , Evolution of Drug Discovery (n. 32), 288–92.

59

DeVitaChu , “History of Cancer Chemotherapy” (n. 30), 8643.10.1158/0008-5472.CAN-07-661118974103

60

GoodmanJordanWalshVivien , The Story of Taxol: Nature and Politics in the Pursuit of an Anti-Cancer Drug (Cambridge: Cambridge University Press, 2001), 12.

61

DeVitaChu , “History of Cancer Chemotherapy” (n. 30), 8643.10.1158/0008-5472.CAN-07-661118974103

62
Ibid., 8645; see also GoodmanWalsh , Story of Taxol (n. 60), 17–19.

63

GoodmanWalsh , Story of Taxol (n. 60), 19.

64

DeVita , “The Drug Development and Clinical Trials Programs of the Division of Cancer Treatment, National Cancer Institute,” Cancer Clin. Trials 2 (1979): 195–216.

65

GoodmanWalsh , Story of Taxol (n. 60), 19–20. These plant-based drugs act by interfering with the microtubule structures in the cells.

66

KeatingPeterCambrosioAlberto , Cancer on Trial: Oncology as a New Style of Practice (Chicago: University of Chicago Press, 2012), 271–76.

67

FanuLe , Rise and Fall (n. 8), 145; PickstoneJohn , “Contested Cumulations: Configurations of Cancer Treatments through the Twentieth Century,” in Cantor , “Cancer in the Twentieth Century” (n. 24), 164–96.10.1353/bhm.2007.0011PMC263584217369667

68

KeatingCambrosio , Cancer on Trial (n. 66), chap. 2.

69

Weatherall , In Search of a Cure (n. 31), chap. 11.

70

ReaderW. J. , Imperial Chemical Industries, vol. 1 (Oxford: Oxford University Press, 1970), 326–27.

71

HounshellDavid A.SmithJohn Kenly , Science and Corporate Strategy: Du Pont R&D, 1902–1980 (Cambridge: Cambridge University Press, 1988), chap. 10.

72 Ibid., 448–49.

73 The term “speculative” referred to a theoretical approach to drug development, based on scientific hypotheses, in contrast to more routine chemical investigations. See AstraZeneca ICI Reports (hereafter AZ) CPR 3, minutes of first meeting of the Chemotherapeutic Research Committee, January 19, 1948.

74 AZ CPR 3, special meeting, March 16, 1953.

75
Reader, Imperial Chemical Industries (n. 70), 92.

76
AZ CPR 3, SpinksA. , “Survey of New Fields for Pharmacological Research,” 3 24, 1953 For more on this, see QuirkeViviane , “From Evidence to Market: Alfred Spinks’s 1953 Survey of New Fields for Pharmacological Research, and the Origins of ICI’s Cardiovascular Programme,” in Medicine, the Market and the Mass Media: Producing Health in the Twentieth Century, ed. BerridgeVirginiaLoughlinKelly (New York: Routledge, 2005), 146–71.

77

KennedyCarol , ICI: The Company That Changed Our Lives (London: Hutchinson, 1986), 130–35; SadoveM. S.WallaceV. E. , Halothane (Oxford: Blackwell, 1962), 3–6.

78
For more on the history of the beta-blockers, see QuirkeViviane , “Putting Theory into Practice: James Black, Receptor Theory, and the Development of the Beta-Blockers at ICI, 1958–1978,” Med. Hist. 50 (2006): 69–92.1650287210.1017/s0025727300009455PMC1369014

79 Ibid.

80 AZ DO 759, H.O. Intelligence Dept., May 1, 1960, Organigram of ICI’s Pharmaceutical Division in 1960; AZ DO 770, Director’s Secretary’s Dept., May 1965, Organigram of ICI’s Pharmaceutical Division in 1965.

81 AZ PH 15355B, Inderal Development Programme, June 15, 1964.

82
For a study of the impact of diuretics, which paved the way for the use of beta-blockers in hypertension, on the understanding of high blood pressure as a disease, see Greene , Prescribing by Numbers (n. 1), chap. 2.

83

HollandH. , “IC Pharmaceuticals,” Pharmaceut. J. (9 12, 1987): 286–88.

84

ChabnerRoberts , “Chemotherapy and the War on Cancer” (n. 28), 70 For a cost–benefit analysis of cancer drug innovation, see, e.g., LichtenbergFrank , “Pharmaceutical Innovation and U.S. Cancer Survival: Evidence from Linked SEER-MEDSTAT Data,” Forum Health Econ. Policy 10 (2007): 1–24.

85

WatkinsonHaddowPaterson , “Influence of Synthetic Oestrogens” (n. 42); WalpoleA. L.PatersonE. , “Synthetic Oestrogens in Mammary Cancer,” Lancet 254 (1949): 783–89.10.1016/s0140-6736(49)91370-715391512

86

CarringtonH. C.CrowtherA. F.DaveyD. G.LeviA. A.RoseF. L. , “A Metabolite of Paludrine with High Antimalarial Activity,” Nature 168 (1951): 1080.1491064310.1038/1681080a0

87

SucklingC. W.LangleyB. W. , “Francis Leslie Rose,” Biog. Mem. Fell. Roy. Soc. 36 (1990): 491–524, 507–8.11616178

88
Since the 1930s it was known that viruses can induce tumors in laboratory mice. The oncogenic potential of a number of virus groups, including adenoviruses, herpesviruses, and poxviruses, was identified in the 1950s and 1960s. See RigbyP. W. J.WilkieN. M. , “Editors’ Preface,” in Viruses and Cancer, ed. RigbyP. W. J.WilkieN. M. (Cambridge: Cambridge University Press, 1985), n.p.

89

PietersToine , Interferon: The Science and Selling of a Miracle Drug (London: Routledge, 2005), esp. chaps. 5–6.

90 AZ CPR 70, Oral Contraception.

91 Patents UK 101397, UK 1064629.

92 AZ PH 19597 B, Nolvadex Development Programme, June 1971.

93 Ibid.

94

JordanV. C. , “Tamoxifen: A Most Unlikely Pioneering Medicine,” Nature Rev. Drug Discov. 2 (2003): 205–13; see also QuirkeViviane , “Standardising Pharmaceutical R&D in the second Half of the Twentieth Century: ICI’s Nolvadex Development Programme in Historical and Comparative Perspective,” in Harmonizing Drugs: Standards in 20th-Century Pharmaceutical History, ed. BonahChristianMasuttiC.RasmussenA.SimonJ. (Paris: Glyphe, 2009), 123–50.10.1038/nrd103112612646

95

ColeM. P.JonesC. T. A.ToddI. D. , “A New Anti-Oestrogenic Agent in Late Breast Cancer. An Early Appraisal of ICI46474,” Brit. J. Cancer 25 (1971): 270–75.511582910.1038/bjc.1971.33PMC2008453

96
These suspicions may have been due to a 1971 report in JAMA, which had suggested that there was a link between diethylstilbestrol and a rare form of vaginal cancer. This was promptly followed by an FDA bulletin warning against the use of DES. See U.S. Food and Drug Administration (FDA), “Certain Estrogens for Oral or Parenteral Use. Drugs for Human Use; Drug Efficacy Study Implementation,” Fed. Regist. 36, no. 217 (1971): 21537–38.

97
AZ PH 27039 B, RichardsonDN , “The History of Nolvadex,” 5 13, 1980; see also Jordan , “Tamoxifen: A Most Unlikely Pioneering Medicine” (n. 94).

98 AZ PH 27039 (n. 97).

99 Indeed, even as late as September 1982, at the annual portfolio review attended by the managers of the Biology (Dr. J. D. Fitzgerald) and Chemistry (Dr. R. Clarckson) Departments, the manager of the Marketing Department, who also attended the meeting, commented that “there was no market for cancer.” Dr. J. D. Fitzgerald, personal communication, November 1, 2013.

100 AZ PH 27039 (n. 97).

101 AZ CPR 110 (1965–77), Cell Division and Growth.

102
On Alderley Park, see AstraZeneca, “Alderley Park, Cheshire,” http://www.astrazeneca.co.uk/astrazeneca-in-uk/our-uk-sites/alderley-park (accessed November 2, 2013). For news on the recent relocation of the firm’s UK R&D center to Cambridge, see http://www.telegraph.co.uk/finance/newsbysector/pharmaceuticalsandchemicals/9937889/AstraZeneca-moves-HQ-to-Cambridge-in-research-revamp.html (accessed November 2, 2013).

103

JordanV. C.CollinsM. M.RowsbyL.PrestwichG. , “A Monohydroxylated Metabolite of Tamoxifen with Potent Antioestrogenic Activity,” J. Endocrinol. 75 (1972): 289–98.10.1677/joe.0.0750305591813

104
Nolvadex Adjuvant Trial Organization, “Controlled Trial of Tamoxifen as Adjuvant Agent in Management of Early Breast Cancer,” Lancet 321 (1983): 257–61; Nolvadex Adjuvant Trial Organization, “Controlled Trial of Tamoxifen as A Single Adjuvant in Management of Early Breast Cancer,” Brit. J. Cancer 57 (1988): 608–11.6130291

105
“Early Breast Cancer Trialists’ collaborative Group. Systemic Treatment of Early Breast Cancer by Hormonal, Cytotoxic, or Immune Therapy. 133 Randomised Trials Involving 31,000 Recurrences and 24,000 Deaths among 75,000 Women,” Lancet 339 (1992): 1–15; “Early Breast Cancer Trialists’ collaborative Group. Tamoxifen for Early Breast Cancer: An Overview of the Randomised Trials,” Lancet 351 (1998): 1451–67. The FDA approved tamoxifen for the reduction of breast cancer risk in 1998. See also JordanV. C. , “Tamoxifen (ICI 46,474) as a Targeted Therapy to Treat and Prevent Breast Cancer,” Brit. J. Pharmacol. 147 (2006): S269–76, S272; LöwyI. , “Treating Health Risks or Putting Healthy Women at Risk: Controversies around Chemoprevention of Breast Cancer,” in GaudillièreHess , Making Drugs (n. 19), 206–27.

106

ChauveauSophie , L’Invention pharmaceutique: la pharmacie française entre l’Etat et la société au Xxe siècle (Paris: Institut d’Edition Sanofi-Synthélabo, 1999), 577; CayezPierre , Rhône-Poulenc, 1895–1975 (Paris: Armand Colin, 1988), 121.

107

Cayez , Rhône-Poulenc (n. 106), 126; SlinnJudy , A History of May & Baker, 1834–1984 (Cambridge: Hobsons, 1984), chap. 5.

108

LiebenauJonathanRobsonMike , “L’Institut Pasteur et l’industrie pharmaceutique,” in L’Institut Pasteur: contributions à son histoire, ed. MorangeMichel (Paris: La Découverte, 1991), 52–61; QuirkeViviane , collaboration in the Pharmaceutical Industry: Changing Relationships in Britain and France, 1935–1965 (London: Routledge, 2008), chaps. 1 and 2.

109

RobsonMichael , “The French Pharmaceutical Industry, 1919–39,” in Pill Peddlers: Essays on the History of the Pharmaceutical Industry, ed. LiebenauJ.HigbyG. J.StroudE. C. (Madison, Wis.: American Institute of the History of Pharmacy, 1990), 107–22; Blondel-MégrelisMarika , “La pharmacie en France 1900–1950, points de repères,” in Les Sciences biologiques et médicales en France, 1920–1950, ed. DebruC.GayonJ.PicardJ.-F. (Paris: CNRS, 1994), 283–96.

110
See QuirkeViviane , “War and Change in the Pharmaceutical Industry: A Comparative Study of Britain and France in the Twentieth Century,” in “Industries du médicament et du vivant,” ed. ChauveauSophie , Enterprises et Histoire 36 (2004): 64–83.

111

HalpernBernard , “Les Antihistaminiques de synthèse. Essais de chimiothérapie des états allergiques,” Archives Internationales de Pharmacodynamie et de Thérapie 68 (1942): 339–408.

112

Chevassus-au-LouisNicolas , Savants sous l’Occupation: enquête sur la vie scientifique française entre 1940 et 1944 (Paris: Seuil, 2004); Quirke , Collaboration in the Pharmaceutical Industry (n. 108), chaps. 4–5.

113

SourkesT. L. , “1957 Daniel Bovet,” Nobel Prize Winners in Medicine and Physiology, 1901–1965 (London: Abelard and Schuman, 1966), 342–51.

114
A brief allusion to Rhône-Poulenc’s antimalarial program can be found in Weatherall , In Search of a Cure (n. 31), 192.

115

ViaudP. , “Les amines dérivées de la phénothiazine,” Journal de Pharmacie et Pharmacologie 6 (1954): 361–89.13184413

116 Rhône-Poulenc Santé archive (hereafter RPS) 10003.

117

GambrelleF. , Innovating for Life: Rhône-Poulenc, 1895–1995 (Paris: Albin Michel, 1995), 44–49.

118

CaldwellA. E. , Origins of Psychopharmacology: from CPZ to LSD (Springfield, Ill.: Charles C Thomas, 1970), 38.

119

DelayJ.DenikerP. , Méthodes chimiothérapiques en psychiatrie: les nouveaux médicaments psychotropes (Paris: Masson, 1961).

120 Laborit went to America accompanied by Deniker in 1953, at a time when Rhône-Poulenc’s marketing campaign extended to the United States. RPS 10003.

121
Much of the history that follows is based on SwazeyJudith , Chlorpromazine in Psychiatry: A Study of Therapeutic Innovation (Cambridge, Mass.: MIT Press, 1974), chap. 7.

122 Ibid., 158–60.

123 Ibid., 164–65.

124 Ibid., 173.

125 Ibid., 161.

126 Boyer, quoted in ibid., 161.

127

Gambrelle , Innovating for Life (n. 117), 49; Cayez , Rhône-Poulenc (n. 106), pt. 2.

128
For more on the pivotal role played by war in the history of Rhône-Poulenc, see Quirke , “War and Change” (n. 110). See also CayezP. , “Négocier et survivre: la stratégie de Rhône-Poulenc pendant la seconde guerre mondiale,” Histoire, Économie et Société 3 (1992): 479–92.

129
RPS 10285, “Visite des Drs Major et Molitor” (20 Mai 1948); RPS 10285, “Entretien avec Mr Georges de Merck” (13 Mai 1949).

130

EndicottK. M. , “The Chemotherapy Program,” J. Nat. Cancer Inst. 19 (1957): 257–93.13502717

131

Weatherall , The Search for a Cure (n. 31), chap. 11; Sneader , Drug Discovery (n. 33), 311–13.

132

QuirkeViviane , “From Antibiotics to Cancer Chemotherapy (1950s–1980s): The Transformation of Rhône-Poulenc in the Era of Biomedicine,” in “Circulation of Antibiotics: Journeys of Drug Standards, 1930–1970,” ed. RomeroA.GradmannC.SantesmasesM. (ESF Networking Program DRUGS Preprint no. 1, Madrid and Oslo, May 2010), 161–74.

133
Much of this information is based on a document produced in 1976, describing Rhône-Poulenc’s approach to cancer chemotherapy. Sanofi-Aventis (hereafter SA) 894402B24, DuboscM. M. , “Cancer Research at the Rhône-Poulenc Company” (10 22, 1976).

134

Gambrelle , Innovating for Life (n. 117), 34.

135 SA 8944002B29, technical note, September 10, 1973.

136 SA 894403B2, Note R. Maral, October 16, 1973.

137
SA 8944002B29, Note ViaudP.MaralR. , 5 24, 1972.

138 SA 894403B2, Letters Robert S. Benjamin to Dr. René Maral, August 9, 1974, and September 13, 1974.

139 SA 894403B2, R. Panier (ST/Dev) to DRT, April 15, 1975.

140

FoucaultD. , ed., Lutter Contre le Cancer (1740–1960) (Toulouse: Editions Privat, 2012); PicardJean-FrançoisMouchetSuzy , La Métamorphose de la medicine: histoire de la recherche médicale dans la France au XXe siècle (Paris: Presses Universitaires de France, 2009), chaps. 6 and 8.

141

WalshVivienLe RouxMuriel , “Contingency in Innovation and the Role of National Systems: Taxol and Taxotère in the USA and France,” Res. Policy 33 (2004): 1307–27.

142

Gambrelle , Innovating for Life (n. 117), 108–11.

143 SA 907635B14, “RPS dans le marché mondial des anticancéreux.”

144

WalshLe Roux , “Contingency in Innovation” (n. 141).

145
The legacy of companies’ historical trajectories has been referred to and examined as “path dependence.” See DavidP. , “Path Dependence and Predictability in Dynamic Systems with Local Network Externalities: A Paradigm for Historical Economics,” in Technology and the Wealth of Nations: The Dynamics of Constructed Advantage, ed. ForayD.FreemanC. (London: Pinter, 1993), 209–31; David , “Why Are Institutions the ‘Carriers of History’? Path Dependence and the Evolution of Conventions, Organizations and Institutions,” Structural Change Econ. Dynamics 5 (1994): 205–20.

146

CantorDavid , “Introduction: Cancer Control and Prevention in the Twentieth Century,” in Cantor, “Cancer in the Twentieth Century” (n. 24), 1–38 (15).10.1353/bhm.2007.000117369661

147

SmithLisa , “Re-thinking the Medical Marketplace in Early Modern London,” H-Net Rev. Human. Soc. Sci. (11 2004), http://www.h-net.org/reviews/showrev.php?id=10018 (accessed May 4, 2014).

148 Ibid.

149

Jordan , “Tamoxifen (ICI46474)” (n. 105).

